# Natural and Artificial Selection for Parasitoid Resistance in *Drosophila melanogaster* Leave Different Genetic Signatures

**DOI:** 10.3389/fgene.2019.00479

**Published:** 2019-05-31

**Authors:** Sylvia Gerritsma, Kirsten M. Jalvingh, Carmen van de Beld, Jelmer Beerda, Louis van de Zande, Klaas Vrieling, Bregje Wertheim

**Affiliations:** ^1^Evolutionary Genetics, Development and Behaviour, Groningen Institute for Evolutionary Life Sciences, University of Groningen, Groningen, Netherlands; ^2^Plant Cluster, Institute of Biology, Sylvius Laboratory, Leiden University, Leiden, Netherlands

**Keywords:** phenotype–genotype associations, parasitoid resistance, genetic variation, adaptive evolution, linkage disequilibrium

## Abstract

Adaptation of complex traits depends on standing genetic variation at multiple loci. The allelic variants that have positive fitness effects, however, can differ depending on the genetic background and the selective pressure. Previously, we interrogated the *Drosophila melanogaster* genome at the population level for polymorphic positions and identified 215 single nucleotide polymorphisms (SNPs) that had significantly changed in frequency after experimental evolution for increased parasitoid resistance. In the current study, we follow up on 11 of these SNPs as putative targets of the experimental selection process (Jalvingh et al., 2014). We study the patterns of genetic variation for these SNPs in several European field populations. Furthermore, we associate the genetic variation of these SNPs to variation in resistance against the parasitoid *Asobara tabida*, by determining the individual phenotype and SNP genotype for 144 individuals from four Selection lines and four non-selected Control lines and for 400 individuals from 12 Field lines that differ in parasitoid resistance. For the Selection lines we additionally monitored the changes in allele frequencies throughout the five generations of experimental selection. For three genes, *mbl* (Zn-finger protein*), mthl4* (G-protein coupled receptor) and *CG17287* (protein-cysteine *S*-palmitoyltransferase) individual SNP genotypes were significantly associated with resistance level in the Selection and Control lines. Additionally, the minor allele in *mbl* and *mthl4* were consistently and gradually favored throughout the five generations of experimental evolution. However, none of these alleles did appear to be associated to high resistance in the Field lines. We suggest that, within field populations, selection for parasitoid resistance is a gradual process that involves co-adapted gene complexes. Fast artificial selection, however, enforces the sudden cumulating of particular alleles that confer high resistance (genetic sweep). We discuss our findings in the context of local adaptation.

## Introduction

Evolutionary adaptation is the progressive genetic improvement in populations resulting from natural selection (Hartl and Clark, 2007). The ability of a population to adapt to changing environments depends on the standing genetic variation in relevant genes. For complex traits, many loci may contribute to the phenotypic variation that selection can act upon. When selection favors particular alleles, the alleles themselves increase in frequency in the population over time, while genetic variants that co-occur in the vicinity of the allele under selection may also change in frequency due to linkage and genetic hitchhiking (Hartl and Clark, 2007; Nuzhdin and Turner, 2013). Thus, selection can leave a localized “footprint” in the genome, which is characterized by changed patterns of genetic variation in a chromosomal segment including the allele under selection. In population genetics and population genomic analyses, such segments with signatures of selection can be identified by changed levels of heterozygosity, increased *F_ST_* values and increased divergence, compared to either the rest of the genome or to non-selected populations. Identifying these loci can aid in elucidating the genetic mechanisms of phenotypic variation, and help to gain a better insight in the evolutionary processes shaping adaptive traits (Frydenberg et al., 2003; Hartl and Clark, 2007; Paaby et al., 2010).

Experimental selection approaches are used to study adaptive evolutionary processes in a controlled environment. By exposing replicate populations, derived from a single genetically variable source population, to a particular environmental condition, experimental selection mimics a replicated and controlled natural selection process. Combining experimental selection with Next Generation genome Sequencing (NGS) allows for a comprehensive examination of the genomic changes caused by the selection process. In this approach, NGS interrogates the genome for polymorphic positions that changed in allele frequency after experimental selection and are therefore putatively associated with the changed phenotype (a “select and re-sequence” approach). This technique is being used more and more to reveal the genetic variants that consistently change in frequency between Control and Selection lines (e.g., Stapley et al., 2010; Turner et al., 2011; Kawecki et al., 2012; Nuzhdin et al., 2012; Remolina et al., 2012; Jalvingh et al., 2014; Martins et al., 2014; Jha et al., 2015; Burghardt et al., 2018). An advantage of this technique is that it does not use *a priori* expectations based on gene function and may therefore reveal genes that have not yet been associated with the trait under consideration.

It is important to realize, however, that this “select and re-sequence” approach has certain limitations. Firstly, it is in essence a population genomics approach, which associates an overall change in allele frequency in selected populations with the population level change in the phenotype. Individual variation in phenotypes and genotypes are not directly linked in this approach. Secondly, this approach often yields many genomic positions that significantly change in allele frequency, not because they are causal, but rather because they are in linkage disequilibrium with a selected position (Nuzhdin and Turner, 2013). While this aids in finding the chromosomal segments that carry a signature of selection, it complicates the identification of the actual genes that were the target of selection. Additionally, statistics in genomics datasets typically involves millions of SNPs, which often results in false positive findings. Therefore, even though alleles of interest can be attributed to the selection response, it is unclear whether an individual with a particular candidate allele indeed shows the associated phenotype, i.e., whether this locus is causal. Thirdly, experimental evolution is performed in a single genetic background, while genetic background can have large effects on the phenotypic effects of a polymorphism. It only targets loci that are variable in that particular starting population, and genes that affect the trait but are homozygous in the starting populations would not be detected. Additionally, epistasis is pervasive, and combinatorial effects of alleles may vary among different genetic backgrounds (David et al., 2004; Mackay, 2004). Careful following-up on results obtained through genomic studies is therefore imperative in the characterization of the effects of selection.

In a recent ‘evolve and re-sequence’ study, we aimed to reveal the genomic basis of adaptive evolution in parasitoid resistance in *D. melanogaster*. This study applied a fast and strong selection pressure that resulted in a rapid phenotypic response. Some *D. melanogaster* larvae, when infected by the parasitic wasp *Asobara tabida*, are able to mount a successful immune response that results in the encapsulation of the parasitoid egg. Experimental selection changed the frequency of successful encapsulation from approximately 20% in the Control lines to 50% in the Selection lines after only five generations of selection (Jalvingh et al., 2014). Genomic re-sequencing of the four replicate Selection and Control lines identified 24 genomic regions affected by selection, including one region of 600 kb on the right arm of chromosome 2 that was particularly well supported by frequency differences in individual SNPs. This region thus carried a strong signature of selection and was therefore considered to be a putative region of major effect on parasitoid resistance (Jalvingh et al., 2014). In this region allele frequencies of 32 genes had significantly changed in the selected lines. It is, however, not likely that all these genes are functionally related to the trait or were actually targets of selection. Rather, through linkage, some allelic variants that flank the targets of selection could have been swept along through a hitchhiking process (Nuzhdin et al., 2007; Nuzhdin and Turner, 2013). To find the actual targets of selection for parasitoid resistance, we need to analyze genotype–phenotype associations at the individual level.

Untangling the genetic variation within linkage blocks with signatures of selection may also reveal whether similar SNPs are selected in the evolution of parasitoid resistance in lines with different genetic backgrounds. We can exploit the genetic variation in natural populations that differ in parasitoid resistance. Previous studies showed large geographical variation in immunological resistance in *D. melanogaster* against the parasitoid *A. tabida*, with natural populations in southern Europe having a higher resistance than natural populations elsewhere in Europe (Kraaijeveld and van Alphen, 1995; Kraaijeveld and Godfray, 1999; Gerritsma et al., 2013). The presence and patterns of genetic variation in these natural populations may aid in identifying the actual targets of selection. Firstly, sequence analyses can reveal whether the same polymorphisms occurs in different natural populations, and whether these SNPs also carry signatures of selection in these populations. Secondly, chance associations among loci due to physical proximity with alleles favored by selection will have largely disappeared, because the selective forces have been more diffuse and persisted over many more generations, allowing for more recombination and compensatory evolution. Moreover, we can assess for the same polymorphisms whether they are associated with high resistance in different genetic backgrounds.

In this study, we follow up on a selection of candidate SNPs, identified by the “selection and resequence” study of Jalvingh et al. (2014). Our aim is to further elucidate the genetic basis of the response to selection for increased parasitoid resistance, to distinguish between the SNPs that were target of selection and those that are linked by chance associations. Additionally, we want to compare genotype–phenotype associations for the same candidate SNPs in both the experimentally evolved lines and a set of lines from natural populations. Firstly, we assessed the patterns of genetic variation in a set of Field lines for several candidate genes within a genomic region that were affected by experimental evolution in the study by Jalvingh et al. (2014), and determined which polymorphic sites carried any signs of selection in the Field lines. Secondly, we associated individual SNP genotypes with individual resistance ability, to assess which alleles were associated with an increased ability of successful immune response against the parasitoid *A. tabida*, and which were not. Thirdly, we assessed allele frequency change for the SNPs throughout the five generations of selection, to identify which combinations of SNPs increased and which decreased in frequency throughout the selection process. Combined, this approach allowed us to dissect the response to selection, and to associate polymorphisms to individual phenotypic differences for different genetic backgrounds.

## Materials and Methods

### Insect Stocks and Cultures

#### Rearing Conditions

All flies were kept as mass cultures (≫1,000 individuals/line/generation) at 20°C under a dark–light regime of 12:12 in quarter pint bottles containing 30 ml standard medium [26 g dried yeast, 54 g sugar, 17 g agar, and 13 ml of a nipagin solution (10 g/100 ml 96% alcohol) per liter]. All lines were kept at standardized densities of ∼200 flies/bottle.

#### Selection and Control Lines

The stocks and selection regime have been described in detail in Jalvingh et al. (2014). Briefly, four replicate Selection and Control lines were established from a single, outbred, source population (Kraaijeveld and Godfray, 1997). During five generations, second instar *Drosophila melanogaster* larvae were exposed to the parasitoid wasp *Asobara tabida* for 24 h and were allowed to develop into pupae. All pupae were visually checked for the presence of a melanized capsule, indicating an immune response against the parasitoid. Only adult flies emerging from a pupa containing a capsule (i.e., those that survived parasitization) contributed to the next generation. In the Control lines the same number of pupae was collected as in the Selection lines. After five generations of selection parasitoid resistance had increased from 20% resistance in the Control lines to 50% resistance in the Selection lines (Jalvingh et al., 2014). The Selection and Control lines were subsequently maintained without further selection, except for one generation of re-selection six generations after the initial experiment. The samples for the genotype–phenotype assays were collected after 46 generations of no selection. Three of the four Selection lines still had a higher level of resistance to parasitoid infection than the Control lines (GLM, chi-square = 18.791, replicate 1: df = 1, *P* < 0.001; replicate 2: chi-square = 5.9093, df = 1, *P* = 0.015; replicate 3: chi-square = 1.3421, df = 1, *P* = 0.246; replicate 4: chi-square = 7.2352, df = 1, *P* = 0.007).

#### Field Lines

Field lines of *D. melanogaster* were collected from natural populations in Europe in the summer of 2009. These Field lines showed substantial variation in their ability to encapsulate *A. tabida* eggs (see Table 1a for collection sites and the encapsulation rate for each line). The Field lines show considerable levels of genetic differentiation from each other, as indicated by pairwise *F_ST_*/*G_ST_* that were calculated based on genotyping of 12 individual flies per Field line for 16 microsatellite markers (Supplementary Materials and Methods, Supplementary Tables S1–S3). The ability to encapsulate *A. tabida* eggs (encapsulation rate, ER) was measured in each line by dissections of parasitized larvae 96 h post-parasitization. Resistance of each line is expressed as the percentage of parasitized larvae that had fully melanized a parasitoid egg. Full details on collection methods and measurements of resistance against *A. tabida* of the Field lines can be found in Gerritsma et al. (2013).

**Table 1:** Encapsulation rate and sample sizes for genotyping assay of the *D. melanogaster* Field lines, Selection, and Control lines.

		(a) Encapsulation rates (resistance)				(b) N genotyped individuals					
Line	Collection site	2009		Current study		% melanization					
		(Gerritsma et al., 2013)									
		ER (%)	*N*	ER (%)	*N*	0%	1–25%	26–74%^∗^	75–99%	100%	*N* total
BAY	Bayreuth, Germany	3.7	30	3.9	51	17	0	–	7	2	26
STA	St Andrews, Scotland	3.3	27	5.7	35	19	0	–	1	2	22
GRO	Groningen, Netherlands	15.6	32	18.8	48	17	0	–	6	9	32
BRE	Bremen, Germany	25.9	27	12.8	39	17	0	–	3	4	24
INN	Innsbruck, Austria	27.9	22	16.7	30	14	5	–	1	7	27
BER	Berlin, Germany	–	–	22.0	50	11	6	–	9	11	37
AVI	Avignon, France	–	–	22.7	44	14	6	–	5	10	35
ARL	Arles, France	45.5	33	26.1	46	13	5	–	5	12	35
BEA	Beaune, France	–	–	29.4	51	10	7	–	10	13	40
KAL	Kaltern am See, Italy	44.4	27	31.1	45	12	6	–	3	11	32
PAR	Paris, France	–	–	32.7	52	6	6	–	11	15	38
GOTH	Gotheron, France	46.4	28	34.4	61	5	13	–	14	20	52

						155	54	–	75	116	400

**Line**	**Regime**	**2009**		**Current study**							
		**(Jalvingh et al., 2014)**									
		**ER (%)**	***N***	**ER (%)**	***N***	**0%**	**1–25%**	**26–74%^∗^**	**75–99%**	**100%**	***N* total**

C1	Control line 1	21.2	85	9.5	42	8	8	0	1	4	21
C2	Control line 2	19.8	91	0.0	24	1	2	3	0	0	6
C3	Control line 3	22.1	68	17.2	29	1	3	2	2	3	11
C4	Control line 4	18.2	77	29.2	24	4	3	2	1	6	16
S1	Selection line 1	54.2	72	47.9	71	1	12	6	3	24	46
S2	Selection line 2	49.3	73	16.7	24	0	4	1	0	1	6
S3	Selection line 3	56.3	48	30.0	30	0	7	4	3	5	19
S4	Selection line 4	42.9	91	69.6	23	0	1	1	0	17	19

						15	40	19	10	60	144

***(a)** Encapsulation rate (% individuals that successfully encapsulated a parasitoid egg) for each line, measured when the lines were collected or generated in 2009, and measured in the current study, 45–50 generations later. The sample sizes, *N*, refer to the number of individuals scored for encapsulation ability. **(b)** Sample sizes of the phenotyped individuals for the SNP genotyping study. Individuals are categorized according to the percentage melanization present around the parasitoid egg. For the Field lines the category 26–74% melanization was not considered for genotyping (^∗^).*

#### Parasitoids

All infections, both in experimental selection and resistance assays, were achieved by exposing *D. melanogaster* larvae to *A. tabida* parasitoids. The parasitoids were cultured at 20°C under a dark–light regime of 12:12. After eclosion all adult parasitoids were collected and stored at 12°C. The *A. tabida* SOS strain was used for both the experimental selection procedure and the resistance assays of the Selection and Control lines. The SOS strain was originally collected in Sospel, France, and was maintained on *D. subobscura* larvae. For the resistance assays of the Field lines, we used the *A. tabida* TMS strain. TMS was established as an isofemale line in 2010 from a cross between two lines: SOS and another from Pisa (Italy) and has been maintained on *D. melanogaster* larvae (Ma et al., 2013). When the Field lines were originally tested for parasitoid resistance, we used both the SOS and the TMS strain (Gerritsma et al., 2013). Resistance in the Field lines against the SOS and TMS strains is strongly correlated (Spearman’s rank correlation, ρ = 0.83, *P*-value = 0.015; Gerritsma et al., 2013), but TMS is more virulent and results are slightly less variable with this parasitoid strain.

### Genetic Variation in Field Lines for Candidate Genes

We selected a set of candidate genes for parasitoid resistance, located in the 600 kb region on 2R and based on the population genomic study by Jalvingh et al. (2014). To measure the genetic variation in these loci in Field lines, we sequenced sections of seven candidate genes surrounding the SNPs that had changed in allele frequency after experimental evolution for increased parasitoid resistance. We analyzed genetic diversity for these candidate genes, and which SNPs carried a signature of selection in the Field lines.

#### Candidate Genes

To select candidate genes from the evolve and re-sequence study of Jalvingh et al. (2014) for characterization in the Field lines, we first filtered for positions that had changed significantly in frequency in the Selection lines compared to the Control lines (FDR corrected *P*-value < 0.01). Among those, we chose seven candidate genes for sequencing in the Field lines, located in a 600 kb region on chromosome 2R that had been highly supported as a candidate region for parasitoid resistance, which we specifically aim to explore. Genes that were selected for sequencing were chosen based on significance of allele frequency change in the genome study of the Selection and Control lines by Jalvingh et al. (2014), SNP position in the gene and gene annotation. These seven genes showed significant differences in allele frequencies for 14 SNPs between the replicated Control and replicated Selection (Jalvingh et al., 2014). In five of these genes, the polymorphic sites from Jalvingh et al. (2014) are located in the coding region, and for two genes these are located in an intron (see Supplementary Table S4).

To explore whether these putative targets of selection were variable, whether we could distinguish different patterns of genetic variation for the loci and whether any showed signs of being under selection in the Field lines, we sequenced representative regions of these seven selected genes in eight Field populations (BAY, STA, GRO, BRE, INN, ARL, KAL, and GOT; for information about sample location and resistance see Table 1a). The Field lines differed in their ability to encapsulate parasitoid eggs, ranging from high, intermediate to low ability. For each gene, 48 individuals were sequenced (six females per line, 96 sequences), with the exception of *CG17287*, where 96 individual females were analyzed (12 per line). We analyzed the genetic diversity across all sequences in order to characterize the patterns of genetic variation and to identify SNPs for which the allele frequencies do not conform to neutrality in the Field lines.

#### Sequencing of Candidate Genes in Field Lines

DNA was extracted using a high salt protocol without chloroform based on Aljanabi and Martinez (1997). Tissue was homogenized in 400 μl homogenizing buffer (0.4M NaCl, 10 mM Tris-HCl pH 8.0, 2 mM EDTA) followed by addition of 40 μl of 20% SDS and 8.5 μl of 10 mg/ml proteinase K (200 μM final concentration). The samples were incubated (1 h at 55°C), after which 190 μl of 6M NaCl (35 g NaCl saturated in 100 ml MQ) was added and samples were vortexed and centrifuged. The supernatant was transferred to new tubes before an equal volume of ice-cold isopropanol was added and the samples were incubated (1 h at -20°C) and centrifuged. The supernatant was removed and the pellet washed 3× with 70% ethanol, dried and suspended in 20 μl MQ.

Pairs of primers were designed to amplify a region of approximately 500 bp of the gene of interest PerlPrimer v1.1.21 (Marshall, 2004) (See Supplementary Table S4). The extracted DNA was diluted 10 times and the primers were diluted to a working solution of 10 μM for PCR and of 5 μM for sequencing. After amplification of the region of interest with a standard PCR (3 min on 94°C, 35 cycles of 94°C for 25 s, melting temperature for 45 s and 72°C for 45 s, 72°C for 7 min), products were purified from excess primers, dNTPs and polymerases by adding the following reaction mix: 0.08 μl ExoI (exonuclease I, 20 U/μl), 0.12 μl FAP (FastAP thermosensitive alkaline phosphasate, 1 U/μl) and 3.8 μl MQ to 5 μl of the PCR product. This was then heated to 37°C for 30 min to activate the enzymes after which the mix was heated to 80°C for 15 min to deactivate the reaction. The product was sequenced by GATC Biotech, Germany, using single-read Sanger sequencing with a standard protocol (both the forward and reverse sequences were obtained).

Sequence products were aligned and processed in CodonCode Aligner 4.1.1. (CodonCode Corporation^1^). Low quality bases and sequencing errors were manually removed, a consensus sequence created, and the low-quality start sequences trimmed, resulting in fragments of ∼400 bp. Reference transcripts were obtained from FlyBase: FB2012_05 Dmel Release 5.47 to determine coding and non-coding regions, and haplotypes were calculated with PHASE, implemented in DNAsp (Stephens and Donnelly, 2003). Alignments were exported as FASTA files and analyzed in DNAsp v5.10.1 (Librado and Rozas, 2009), calculating haplotype diversity, private haplotypes, population genetic parameters, Tajima’s D and nucleotide diversity (π) (results in Supplementary Tables S5–S7).

To test for signatures of positive or balancing selection we combined all SNPs and analyzed the data using an outlier analysis with *F_dist_* implemented into LOSITAN (Beaumont and Nichols, 1996; Antao et al., 2008). This outlier analysis evaluates the relationship between *F_ST_* and *H_e_* (expected heterozygosity), describing the expected distribution of Wright’s coefficient *F_ST_* versus *H_e_* for neutral markers under the assumption of an island model of migration (Wright, 1931). The expected distribution is used to identify outlier loci that have excessively high or low *F_ST_* values compared to neutral expectations with the observed levels of heterozygosity. Such outlier loci are candidates for being subject to selection. Loci are considered candidates for positive selection when the *F_ST_* value was above the 0.95 probability level, and candidates for balancing or frequency dependent selection when the *F_ST_* value fell below the 0.05 probability level (Beaumont and Nichols, 1996; Antao et al., 2008). The analysis was set to 50,000 simulations with a confidence interval of 0.95 and false discovery rate set to 0.1, eight populations, subsample size of 12 and 116 loci (SNPs) with a simulated mean dataset *F_ST_* of 0.105 and an attempted mean neutral *F_ST_* of 0.107, estimated only on putative neutral loci (all potential outlier loci were removed to compute the mean neutral *F_ST_*).

### Individual Genotype–Phenotype Associations

To associate the variation in resistance to specific alleles, we determined individual genotypes for a subset of selected SNPs (see below) in individually phenotyped larvae taken from the Field lines and from the Selection and Control lines. We genotyped these SNPs in individually phenotyped *D. melanogaster* larvae from the Selection and Control lines and from the Field lines. For each of these SNPs, we associated individual SNP genotypes with individual resistance ability, to assess whether the presence of an allele is associated with an increased ability of a successful immune response against the parasitoid *A. tabida*. These individual genotype–phenotype assays were performed on a total of 400 individuals from 12 Field lines with a range of resistance ability, and on a total of 144 individuals from 4 Selection and 4 Control lines (listed in Table 1b).

#### Resistance Levels

Resistance levels were measured according to the dissection protocol described in Gerritsma et al. (2013). In short, eggs were collected within 1 h of oviposition at 25°C, and thereafter kept at 20°C in groups of 50 individuals per petridish (diameter: 55 mm), containing standard medium and live yeast. Four days after oviposition of the eggs, an *A. tabida* wasp was introduced to the second instar *D. melanogaster* larvae and oviposition behavior of the wasps was observed. Only parasitized larvae were collected for further development, which is assumed to have happened when oviposition lasts at least 10 s (van Alphen and Drijver, 1982). Wasps were replaced after they successfully parasitized 10 larvae, and the total period of larval collections per line was approximately 2 h to minimize variation in larval development. Larvae were allowed to develop at 20°C and dissected to assess the presence of a wasp egg and to score the phenotype. We scored encapsulation success, i.e., whether the larva was susceptible (the wasp egg was not completely melanized) or resistant (a complete capsule was formed around the wasp egg). Larvae were dissected 72–96 h post-parasitization.

For the Field lines, a total of 967 larvae were dissected to score their resistance, of which 552 samples gave reliable and useable phenotypes. For the Control and Selection lines, a total of 247 larvae were dissected to score their resistance, of which 202 gave reliable phenotypes. Per line we aimed to genotype 20 samples from the low resistant group (0–25% melanization around the wasp egg, Table 1b) and 20 samples from the high resistant group (75–100% melanization around the wasp egg, Table 1b). Due to variation in resistance among the lines we did not reach these balanced sample sizes for all lines. Most samples that could not be used did not contain a wasp egg (unsuccessful parasitization).

#### Selection of SNPs for Genotyping

**FIGURE 1:**
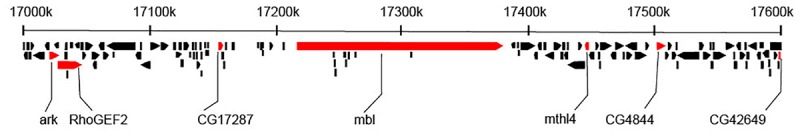
The 600 kb region of chromosome 2R of *Drosophila melanogaster* that was the primary focus of this study. This region was identified as a putative major effect locus for parasitoid resistance in an evolve and re-sequence study (Jalvingh et al., 2014). The figure shows the positions for the genes along the chromosome (based on Flybase version FB2019_01, dos Santos et al., 2014), highlighting the seven genes for which we successfully genotyped a SNP in individuals of Field lines and Selection and Control lines.

In total, we selected 13 candidate SNPs for genotyping that were located in an exon or intron of a gene and had significantly changed in allele frequency in the evolve and re-sequence study (Jalvingh et al., 2014). Three SNPs were chosen based on the *F_ST_*-outlier analysis from the sequencing data on seven gene fragment in the Field lines within the 600 kb region on chromosome 2R that had been highly supported as a candidate region for parasitoid resistance (Jalvingh et al., 2014). These three exceeded the 5–95% probability interval in the *F_ST_*-outlier analysis: two that were potential candidates for positive selection (located in *RhoGEF2* and *mthl4*) and one that was likely under balancing selection (located in *CG42649*). An additional five SNPs were chosen within the same 600 kb region on chromosome 2R (located in *ark, CG17287, CG4844, mbl* and *babos*) (see Figure 1). We chose an additional five non-synonymous SNPs, three located on chromosome 3R (located in *lig3, CG31157* and *CG18765*), one on chromosome 2L (located in *capu*) and one on chromosome 2R (located in *CG34207*). All these SNPs outside the 600 kb region had also changed significantly in allele frequency in the evolve and re-sequence study, but the SNPs on 3R were not associated to a genomic region with a signature of selection, while the other two were each in a different region with a signature of selection (Table 2).

**Table 2:** Overview of the SNPs characterized in this study, including associated gene, Flybase ID, gene annotations and chromosomal location.

Gene	FlyBase ID	Function	SNP name	Chromo some	SNP location	Region (Jalvingh et al., 2014)	SNP	Coding	Minor allele selection
*Ark*	FBgn0024252	ATP binding; contributes to cysteine-type endopeptidase activity involved in apoptotic signaling pathway.	R3_ark_689	2R	12911689	7	C:G	Synonymous	For
*mthl4*	FBgn0034219	G-protein-coupled receptor in stress response	01_mthl4_827	2R	13334827	7	A:G	Non-coding (exon)	For
*RhoGEF2*	FBgn0023172	Rho guanyl-nucleotide exchange factor activity	02_RhoGEF2_160	2R	12930160	7	G:T	Synonymous	For
*CG42649*	FBgn0261501	Gene ontology unknown	03_CG42649_693	2R	13486693	7	C:T	?	For
*CG17287*	FBgn0034202	Zinc finger, DHHC-type, palmitoyltransferase	04_CG17287_210	2R	13045210	7	T:G	Synonymous	For
*CG4844*	FBgn0061355	C-type lectin	05_CG4844_055	2R	13394055	7	C:G	Non-coding (exon)	For
*Mbl*	FBgn0261642	DNA and metal ion binding, apoptosis, cell development and differentiation	06_mbl_670	2R	13159670	7	C:T	Non-coding (intron)	For
*lig3*	FBgn0038035	ATP binding, DNA binding, DNA ligation, recombination repair	07_lig3_427	3R	8225427	–	C:T	Non-synonymous	Against
*CG31157*	FBgn0051157	Gene ontology unknown, possible relation to stress resistance	08_CG31157_443	3R	8874443	–	C:T	Non-synonymous	For
*CG18765*	FBgn0042110	Transferase activity, transferring phosphorus-containing groups	09_CG18765_172	3R	7516172	–	G:C	Non-synonymous	Against
*CG34207*	FBgn0085236	Gene ontology unknown	11_CG34207_969	2R	18216969	8	A:T	Non-synonymous	For

*Each SNP had a significant frequency difference in Selection lines compared to the Control in the genome study, and most were located in genomic regions with a signature of selection (see Jalvingh et al., 2014 for further details).*

During genotyping (see below), the SNPs from the genes *capu* (on 2L) and *babos* (on 2R) showed too many missing or uncalled data points to make any reliable conclusions, and were therefore not analyzed further. The SNP in gene *CG4844* did not show any variation in the Field lines, except for one low resistant individual from the line BRE. This SNP is therefore only analyzed for the Selection and Control lines.

#### Genotyping

Dissected larvae were collected and stored at -20°C in 100 μl TE (1 mM Tris, 0.1 mM EDTA) buffer. For the Field lines DNA was extracted using the same high-salt DNA extraction method as described above under ‘Sequencing of candidate genes.’ For the Selection and Control lines, DNA was extracted using a high-throughput DNA extraction method, adjusted after (Hoarau et al., 2007). Tissue was homogenized with a pestle in 50 μl digestion buffer (2 ml 5M NaCl, 1 ml 1M Tris-HCl pH 8.0, 5 ml 0.5M EDTA pH 8.0, filled up to 97.5 ml with MQ and 2.5 ml 20% SDS added after autoclaving. Another 50 μl of digestion buffer containing 2 μl of 0.4 mg/ml proteinase K was added for overnight incubation at 55°C. After incubation 40 μl of 6M NaCl (35 g NaCl saturated in 100 ml dH_2_O) and 100 μl chloroform was added to each sample. Samples were centrifuged for 20 min at 3,000 rpm and the supernatant transferred to a Millipore (MSFBN6B50) filter plate that contained an equal amount of binding buffer (90.8 g NaI, 1.5 g Na_2_SO_3_, fill to 100 ml with MQ and filter). The filter plate was centrifuged at 1,000 rpm for 15 min and 2,000 rpm for 10 min. The same volume of ice-cold wash buffer (freshly prepared solution of 10.8 ml 100% EtOH and 4.2 ml stock wash buffer: 2.5 ml 4M NaCl, 2 ml 1M Tris-HCL pH 8.0, 0.2 ml 0.5M EDTA pH 8.0, filled up to 100 ml with MQ and autoclaved), was added to the supernatant before centrifuging again for 10 min at 3,000 rpm. The samples were washed three times to remove the high concentration of salt in the samples. The plates were dried at RT during 30 min. DNA was eluted with 100 μl warm (55°C) elution buffer (0.1× TE: 100 μl 1M Tris-HCL pH 8.0, 20 μl 0.5M EDTA, fill up to 100 μl with MQ) and incubated for 5 min before centrifugation (5 min, 1,000 rpm and 5 min 2,000 rpm).

All extracted DNA was diluted and brought to a concentration of 5 ng/μl. All SNPs were genotyped at the Institute of Biology, Leiden University, using a Kompetitive Allele Specific PCR (KASP) genotyping assay (Semagn et al., 2014). For 10 SNP loci KASP primers were designed using the Kraken software of LGC genomics. DNAs were diluted to 1 ng/μl and analyzed in uniplex on the LGC genomics SNP genotyping line according the manufactures’ instructions. Genotypes were called using the Kraken software. Primers were ordered at Integrated DNA Technologies.

#### Statistical Analysis of Phenotype–Genotype Associations

The association of individual SNPs with phenotype was assessed using a Generalized Linear Model (GLM) implemented in R 3.1.2. Specifying a binomial distribution, this model tested the binary response variable Encapsulation success (success or failure) against SNP Genotype (allele 1, allele 2 or heterozygote), Origin (Selection and Control regime or Field line) and the interaction term SNP Genotype:Origin. We used model-simplification, eliminating non-significant variables sequentially from the model.

To compare the extent of linkage disequilibrium in the Control and Selection lines and in the Field lines within the 600 kb region on chromosome arm 2R, we calculated the pairwise linkage disequilibrium for the seven SNPs (six SNPs for the Field lines, because the SNP in *CG4844* was monomorphic in the Field line samples) in the 600 kb region (see Figure 1). Using the R-package “genetics,” version 1.3.8.1 (Warnes et al., 2012), we calculated three pairwise estimators of linkage disequilibrium for all SNPs, based on the individual genotype data (raw D, scaled D′ and the correlation coefficient r), and compared the observed numbers of the various genotypes with the expected numbers based on allele frequencies (as implemented in the R-package genetics with default settings).

### Following SNP Frequencies Through Selection Procedure

We followed the allele frequency change for the SNPs throughout the five generations of experimental selection from samples that were collected during the selection procedure. For this, adult flies that had survived parasitization were sampled throughout the selection process and were individually genotyped. For the SNPs in the well-supported region on chromosome arm 2R we also assessed the linkage phase disequilibrium, identifying which combinations of SNPs increased and which decreased in frequency throughout the selection process.

Throughout the selection process, adults were collected and frozen at -20°C directly after egg-laying. At the onset of selection the source population laid four batches of eggs over 10 days, from each of which one pair of Control and Selection lines was founded. A random subset of the source population was taken immediately after egg-laying for each of these batches. Throughout the five generations of selection, adults of each of the Control and Selection lines were collected and frozen immediately after egg-laying.

DNA was isolated from 24 females per line after 0 (source population), 1, 2, 3, and 5 generations of selection. The tissue of individual flies was homogenized with a motorized pestle and DNA from the adult flies was isolated using the Qiagen DNeasy 96 Blood and Tissue kit, following the Qiagen Purification of Total DNA from Animal Tissues protocol on 96 well plates. Determination of the genotype for each SNP was done at Leiden University as described above.

Linkage phase disequilibrium was scored of six selected SNP positions within a 600 kb region on chromosome arm 2R. We calculated linkage phase disequilibrium in PHASE 2.1.1 (Stephens et al., 2001; Stephens and Donnelly, 2003), specifying a recombination model and initializing estimates for recombination rates between the alleles. Frequency estimates of each phase were calculated by PHASE for each sample-group, and are reported as population linkage phase (from hereon referred to as “haplotype”) frequency. In the PHASE analysis, the generation number (0, 1, 2, 3 or 5) and treatments (Selection or Control regime) were combined to create 10 groups. Per treatment, replicate lines were pooled to increase power in the analysis.

## Results

### Genetic Variation in Field Lines for Candidate Genes

In a previous study, we combined experimental selection for increased parasitoid resistance with whole-genome sequencing, and identified 94 variants within a 600 kb region that had changed significantly (FDR corrected *P-*value of <0.01) in allele frequency in the Selection lines compared to the Control lines (Jalvingh et al., 2014). To identify which of these SNPs may have been the target(s) of selection, we first assessed the presence and patterns of polymorphisms in these loci across eight Field lines.

We sequenced fragments of seven genes containing 14 of these highly significant SNPs. In total we found 116 polymorphic sites in all sequenced fragments of a total of 356 individuals (712 sequences) across the seven candidate genes. This included all 14 SNPs that had been found by Jalvingh et al. (2014). No significant genetic differentiation was found among the lines across all the 116 SNPs (Supplementary Table S5). Average nucleotide diversity across all gene fragments was 2.3 ± 0.4% for synonymous substitutions and 0.13 ± 0.05% for non-synonymous substitutions (Supplementary Tables S6, S7). Expected heterozygosity levels did not differ from observed heterozygosity levels for all genes (Supplementary Table S6, Mann–Whitney *U* test, *p* = 0.971). Yet, we distinguished different patterns of genetic variation for the different loci. Tajima’s D values ranged from significantly negative for *RhoGEF2* to significantly positive for *CG6568*, suggestive or positive or purifying selection, respectively, balancing selection (Supplementary Tables S6, S7). Average haplotype diversity exceeded 50% for all genes, with all genes having one or two common haplotypes and a number of rare ones. The genetic diversity differed among the seven candidate genes, with *mthl4* being the most variable and *RhoGEF2* and *CG42649* showing the least diversity (Supplementary Tables S6, S7). The number of haplotypes ranged from 9 (in *RhoGEF* and *CG6568*) to 27 (in *mthl4*), and the haplotype diversity differed significantly among the different genes (glm, *F* = 9.17, df = 6, *P* < 0.0001). For further information on genetic diversity measurements on these sequenced gene fragments, we refer to Supplementary Tables S5–S7. Thus, these patterns on genetic diversity show differences among the seven gene fragments located in the 600 kb region on 2R, which may suggest they possibly evolved under different types and strengths of selection.

**FIGURE 2:**
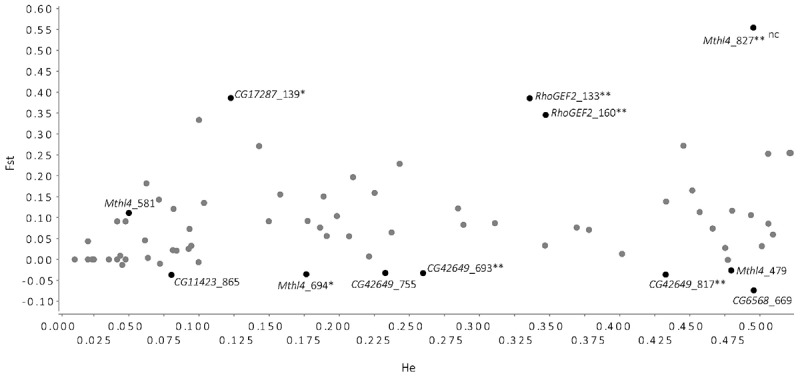
*F_ST_* outlier analyses using FDIST2 method (Beaumont and Nichols, 1996) implemented in LOSITAN (Antao et al., 2008), based on 116 SNPs found in the sequences of the candidate genes in the *D. melanogaster* Field lines. This analysis identifies SNPs with excessively high or low *F_ST_* values (calculated across all the Field lines) that fall outside the expected distribution of *F_ST_* values for expected heterozygosity (*H_e_*) levels, under the assumption of an island model of migration for neutral markers (Wright, 1931). The SNPs that fall within the 5–95% probability interval for the expected distribution of *F_ST_* values are represented by gray dots, indicating putative neutral SNPs and are not labeled. SNPs that fall above the 95% confidence intervals have higher than expected *F_ST_* values and are possible candidates for being under positive selection; SNPs that fall below the 95% confidence interval are likely candidates for being under balancing selection. Black dots represent SNPs that exceed the 5–95% probability interval, and are labeled with the SNP ID and with annotations (nc, non-coding; ^∗^non-synonymous change; ^∗∗^SNP that significantly changed in allele frequency in Selection and Control lines, see Jalvingh et al., 2014).

To evaluate which SNPs (of the 116 in total) showed signatures of any form of selection in the field lines, an *F_ST_* outlier analysis was conducted. Of the SNPs with the most extreme *F_ST_* values compared to neutral expectations with the observed levels of heterozygosity, exceeding the 0.05–0.95 probability interval, two were considered outliers for positive selection and two were considered outliers under balancing selection (FDR < 0.1, Figure 2). The 12 SNPs outside the 5–95% were located in six out of the seven candidate genes. The remaining 104 SNPs did not show evidence of being under any form of selection and are likely to be neutral, based on this outlier test.

The SNPs that showed significant allele frequency differences in the study by Jalvingh et al. (2014) are significantly over-represented in the group of SNPs outside the 5–95% interval (hypergeometric test, *P* < 0.001). Five of the 14 SNPs that also had significantly changed in Jalvingh et al. (2014) fell outside the 5–95% interval (three being candidates for SNPs under positive selection, two under balancing selection). This suggests that the SNPs that were affected by experimental evolution for increased resistance are also those that have most extreme *F_ST_* values, compared to neutral expectations, in natural populations.

### Individual Genotype–Phenotype Associations

#### Selection and Control Lines

We scored the ability of individual *D. melanogaster* larvae from Selection and Control lines to resist parasitization by *A. tabida* (Table 1) and determined their genotype of 11 candidate SNPs (described in Table 2 and see Supplementary Data Sheet for the raw data). We subsequently assessed the association of each SNP to the phenotype in statistical models, evaluating Encapsulation success (success or failure) against SNP Genotype (allele 1, allele 2 or heterozygote), Origin (Selection and Control regime) and the interaction term (see “Materials and Methods” section for details). For all SNPs, the selection regime contributed significantly to explaining the Encapsulation success. This reflects the larger number of resistant individuals in the Selection lines in the assay, and inclusion of ‘Origin’ in the model therefore statistically corrects for this confounding factor.

For three SNPs, in the genes *Muscleblind* (*mbl*), *CG17287* and *mthl4*, we found that allelic variation (‘SNP Genotype’) was significantly associated to resistance (Table 3b). This indicates that either the alleles of these SNPs, or those of a closely linked genetic variant, correlates to parasitoid resistance in these lines. In none of the models we found a significant interaction between SNP genotype and Selection or Control treatment. This suggests that any effects of these SNPs are not conditional on the genetic background present in the Selection and Control lines.

**Table 3:** Statistical analysis of the association between genotype and parasitoid resistance.

		(a) Field lines					(b) Selection and Control lines				
SNP ID	df	Deviance	Resid. df	Resid. Dev	Pr(>Chi)	df	Deviance	Resid. df	Resid. Dev	Pr(>F)	
01_*mthl4*_827				388	468.81						
	Genotype	2	1.52	386	467.29	0.4673	2	9.75	139	183.68	**0.008^∗∗^**
	Origin	11	23.78	375	443.51	**0.0137**^∗^	1	8.17	138	175.51	**0.004^∗∗^**
	Genotype:Origin	20	20.30	355	423.21	0.4394	2	3.14	136	172.37	0.208
02_*RhoGEF2*_160				392	473.34				141	192.78	
	Genotype	2	1.40	390	471.95	0.4967	2	0.17	139	192.61	0.918
	Origin	11	20.41	379	451.53	**0.0400^∗^**	1	13.20	138	179.41	**<0.001^∗∗∗^**
	Genotype:Origin	12	24.02	367	427.52	**0.0202^∗^**	2	0.26	136	179.15	0.878
03_*CG42649*_693				398	481.04				143	195.61	
	Genotype	2	0.70	396	480.33	0.7033	2	1.33	141	194.27	0.513
	Origin	11	20.80	385	459.53	**0.0355^∗^**	1	11.32	140	182.96	**<0.001^∗∗∗^**
	Genotype:Origin	15	8.71	370	450.82	0.8923	2	0.04	138	182.91	0.980
04_*CG17287*_210				396	479.66				142	194.53	
	Genotype	2	1.31	394	478.35	0.5205	2	11.18	140	183.34	**0.004^∗∗^**
	Origin	11	21.50	383	456.85	**0.0285^∗^**	1	8.69	139	174.65	**0.003^∗∗^**
	Genotype:Origin	15	19.43	368	437.42	0.1948	1	0.02	138	174.63	0.902
05_*CG4844*_055^∗^				–	–				141	193.43	
	Genotype	–	–	–	–	–	2	3.54	139	189.89	**0.170**
	Origin	–	–	–	–	–	1	7.96	138	181.93	**0.005^∗∗^**
	Genotype:Origin	–	–	–	–	–	2	0.87	136	181.06	0.647
06_*mbl*_670				395	477.19				142	193.85	
	Genotype	2	0.05	393	477.14	0.9756	2	6.53	140	187.31	**0.038^∗∗^**
	Origin	11	19.70	382	457.44	**0.0497^∗^**	1	6.66	139	180.65	**0.010^∗^**
	Genotype:Origin	19	30.97	363	426.47	**0.0406^∗^**	2	0.95	137	179.70	0.622
07_*lig3*_427				396	474.24				124	169.03	
	Genotype	2	0.48	394	473.76	0.7884	2	4.68	122	164.35	**0.096**
	Origin	11	22.97	383	450.79	**0.0178^∗^**	1	6.84	121	157.51	**0.009^∗∗^**
	Genotype:Origin	6	10.41	377	440.38	0.1084	2	0.87	119	156.64	0.646
08_*CG31157*_443				363	442.66				130	176.80	
	Genotype	2	1.33	361	441.32	0.5134	2	2.10	128	174.70	0.350
	Origin	11	18.47	350	422.86	0.0714	1	11.17	127	163.54	**0.001^∗∗∗^**
	Genotype:Origin	21	16.17	329	406.69	0.7603	2	0.12	125	163.41	0.940
09_*CG18765*_172				393	477.57				141	193.43	
	Genotype	2	0.09	391	477.49	0.9578	2	0.91	139	192.52	0.635
	Origin	11	22.71	380	454.77	**0.0194^∗^**	1	11.41	138	181.12	**<0.001^∗∗∗^**
	Genotype:Origin	13	12.52	367	442.26	0.4858	2	2.63	136	178.49	0.269
11_*CG34207*_969				396	476.07				143	195.61	
	Genotype	1	0.17	395	475.90	0.6807	2	2.65	141	192.96	0.266
	Origin	11	23.55	384	452.35	**0.0148^∗^**	1	9.39	140	183.56	**0.002^∗∗^**
	Genotype:Origin	6	8.90	378	443.45	0.1795	2	0.57	138	183.00	0.754
R3_*ark*_689				392	473.34				137	186.38	
	Genotype	2	2.86	390	470.48	0.2389	2	1.56	135	184.82	0.457
	Origin	11	21.28	379	449.21	**0.0306^∗^**	1	11.55	134	173.26	**0.001^∗∗∗^**
	Genotype:Origin	22	30.09	357	419.12	0.1164	2	0.55	132	172.71	0.758

*To test the effect of genotype on parasitoid resistance we specified a GLM using a binomial distribution, testing the effects of genotype (homozygous for allele 1, homozygous for allele 2, heterozygous), Origin (Field line, Selection or Control regime) and their interaction for each SNP position separately. Significance was determined by model-simplification, eliminating non-significant variables sequentially from the model. The analysis was performed separately for **(a)** the 12 Field lines and **(b)** the two regimes for the Selection and Control lines. ^∗^The SNP in gene *CG4844* did not show any variation in the Field lines, except for one low resistant individual from the line BRE. This SNP is therefore only analyzed for the Selection and Control lines.*

**FIGURE 3:**
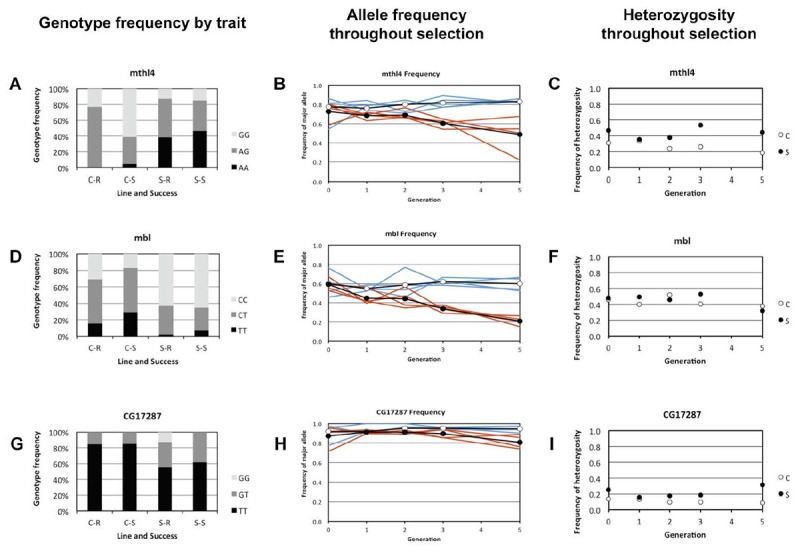
Genotype, allele, and heterozygosity frequencies for the genes that showed a significant association between genotype and resistance ability in the Selection and Control lines. Control lines are in blue and the Selection lines in red. Panels **(A,D,G)** show the depict genotype frequencies in resistant and susceptible larvae of the Selection and Control lines for *mbl, mthl4* and *CG17287*, respectively. For both the Selection (S) and the Control (C) lines, the genotype frequencies are shown separately for resistant (R) and susceptible (S) larvae. Panels **(B,E,H)** show the frequency of the major allele for each generation of selection, pooling the four replicate lines for each selection regime. Open circles denote the average of the control treatment and closed circles the average of the selection treatment. Separate trajectories for these alleles are shown for each Selection line (in red) and Control line (in blue). Panels **(C,F,I)** show the frequency of heterozygotes in each generation of selection (pooled per selection regime).

In *mthl4*, the A allele increased in frequency in the Selection lines, and the G allele decreased strongly in frequency. In the Control lines, most susceptible individuals had the GG genotype, while most resistant individuals were heterozygote. The Selection lines consisted mostly of heterozygotes and individuals with the AA genotype (Figure 3A,B). This fits the expected pattern of selection acting for a dominant allele that contributes to parasitoid resistance and/or to heterozygote advantage.

In *mbl*, the frequency of the T allele decreased in the Selection lines, and the C allele increased in frequency (Figure 3D,E). The TT genotype was more often present in susceptible than in resistant larvae of the Control lines, and the CC genotype was more frequent among the resistant larvae (Figure 3D). In the Selection lines the TT and the CT genotype was found at a much lower frequency than in the Control lines. This fits the expected pattern of selection acting for a minor allele that contributes to parasitoid resistance.

In *CG17287* the G allele increased in the Selection lines and the T allele decreased in frequency (Figure 3G,H). In the Control lines the different genotypes seems to be equally frequent in the resistant and the susceptible larvae, and the GG genotype was only found among the resistant larvae of the Selection line (Figure 3G). The change in allele frequency was relatively small, and fluctuated among the replicate Selection lines. Also, the equal distribution of genotypes among the resistant and susceptible larvae of the Control line is not intuitively clear if this SNP would be causal. This fits the expected pattern of selection acting for a recessive allele that contributes to parasitoid resistance, and with patterns of genetic drift.

#### Field Lines

The percentage of individuals with a fully melanized capsule around the wasp egg differed significantly among lines, ranging from 3.9 to 34.4% (GLM, X_11_= 35.67, *P* < 0.001) (Table 1b, “current study”). Eleven SNPs were genotyped in 400 individually phenotyped larvae to test for associations between genotype and phenotype within lines (see Supplementary Data Sheet for the raw data). Also here, “Origin” had a significant effect on the level of resistance in all genes (Table 3a), reflecting the collection bias in sample sizes for the resistant and susceptible larvae for some of the lines (Table 1b).

Genotype did not explain a significant part of the variation in encapsulation success in the phenotyped individuals across lines for any of the 11 SNPs (Table 3a). For the SNPs in *RhoGEF2* and *mbl*, however, we found a significant interaction between SNP genotype and field line, indicating that per line, genotype had a different correlation to phenotype. Furthermore, more heterozygous individuals for the SNP in *RhoGEF2* were present in the higher resistant lines. Yet, for none of the SNPs was heterozygosity significantly associated to the individual ability to encapsulate wasp eggs. To check whether an allele was correlated to encapsulation success within a line, we selected a subset of four SNPs that showed the highest allele frequency differences between susceptible and resistant individuals within a line (*ark* and *RhoGEF2* in ARL; *mbl* in BRE; *mthl4* in GOTH). For none of these SNPs, we found that individuals carrying a particular allele were more resistant than individuals carrying the alternative allele for that particular SNP. Moreover, when we would correct for the multiple testing (i.e., associations between the genotype for 10 SNPs with individual encapsulation success in the same dataset) across all the Field lines, none of the significant interactions would pass significance. This indicates that the polymorphisms we chose to genotype were not consistently associated with higher parasitoid resistance among or within the Field lines.

#### Linkage Disequilibrium

To assess whether the seven SNPs in the 600 kb region on chromosome 2R (see Figure 1) were in linkage disequilibrium (LD) in the Control and Selection lines and in the Field lines, we calculated pairwise estimators of LD for all SNPs based on the individual genotype data (see Supplementary Figure S1; note that the SNP in *CG4844* was monomorphic in the Field lines and was excluded from the LD calculations for the Field lines).

**FIGURE 4:**
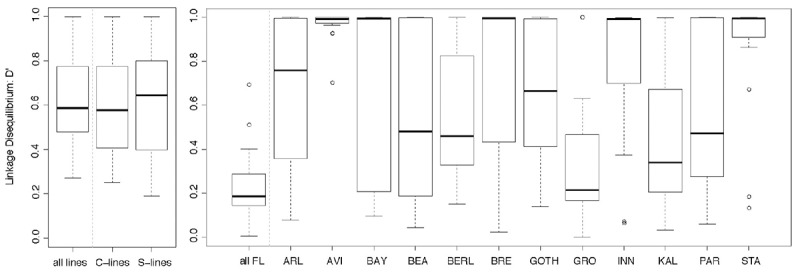
Pairwise estimates of linkage disequilibrium (D′) for six to seven SNPs in a 600 kb region on chromosome 2R. The SNPs that were genotyped are shown in Figure 1; CG4844 was not polymorphic in the Field lines and was therefore excluded from the LD analysis for the Field lines. LD was calculated for the Selection and Control lines (left panel) and for a set of 12 Field lines (right panel). The boxplots represent the distribution of all pairwise LD estimates (D′) between the six to seven SNPs for the individuals that were analyzed for individual genotype–phenotype associations. In the left panel, LD estimates were calculated for the combined Selection and Control lines (*n* = 144), and separately for the pooled Selection lines (*n* = 90) and pooled Control lines (*n* = 54). In the right panel, LD estimates were calculated for the combined Field lines (“all FL,” *n* = 400) and for each Field line separately (*n* = 23–52 per Field line, see Table 1 for abbreviations and sample sizes of the Field lines).

The LD in the Selection and Control lines was highly significant among most pairwise SNPs (*P* < 0.001, Supplementary Figure S1), with slightly higher D′ values in the Selection lines than in the Control lines (Figure 4). In addition, the pairwise disequilibrium plots showed a wider region of high LD in the Selection than in the Control lines, especially in the central region of the 600 kb region. In the Control lines, the SNP in *mbl* showed high LD with the SNP in *CG17287*, which was maintained in the Selection lines while in these Selection lines, the region with high D′ values extended to include *ark, RhoGEF2* (to the left of *mbl*) and *mthl4* (to the right). These patterns of increasing levels of LD and expansion of high LD over a broader region in the Selection lines are consistent with rapid and strong positive selection for an allele within this linkage block. Using PHASE to infer haplotypes for the three SNPs in the middle of the 600 kb region (*CG17287, mbl, mthl4*), the haplotypes GCA and TCA were the most prevalent in the Selection lines, and three to four times more frequent than in the Control lines.

Also in the 12 Field lines combined, there was significant LD among most pairwise SNPs (Supplementary Figure S1). Across the combined Field lines, the highest LD values were mostly restricted to the directly adjacent SNPs (Supplementary Figure S1), and the estimates of the pairwise D′ values were considerably lower than for the Control and Selection lines (Figure 4). However, each individual Field line showed similar or even higher estimates for LD than the Control and Selection lines (Figure 4). The Field lines differed from each other for the most common haplotypes. Some haplotypes were more common across several lines, both when inferring haplotypes for the six SNPs (CTTCAG, GTTCGG, GTTCAG) and for the three most central SNPs (TCA and TCG) in the 600 kb region.

### Following SNP Frequencies Through Selection Procedure

We determined the allele and genotype frequency of each SNP in the parental population and after each of the five generations of selection (Figure 3B,E,H). For two of the SNPs that were significantly associated to parasitoid resistance in the Selection and Control lines (*mbl* and *mthl4*), selection consistently increased the frequency of the minor alleles, causing gradually increasing frequency differences in each generation of selection. This pattern was apparent in all four replicated Selection lines. In the third SNP that was significantly associated to resistance (*CG17287*), the allele frequencies remained fairly constant, except for the last generation in the Selection line. Heterozygosity was not shifted toward an excess or shortage of heterozygotes in *mbl, mthl4* or *CG17287* (Figure 3C,F,I). This indicates that heterozygotes were not disproportionally over- or under-represented among the individuals that survived parasitization. We therefore see no evidence of heterozygote advantage acting on these SNPs during the selection process nor inbreeding, but there is evidence for directional selection for the minor alleles, especially in *mbl* and *mthl4*.

**FIGURE 5:**
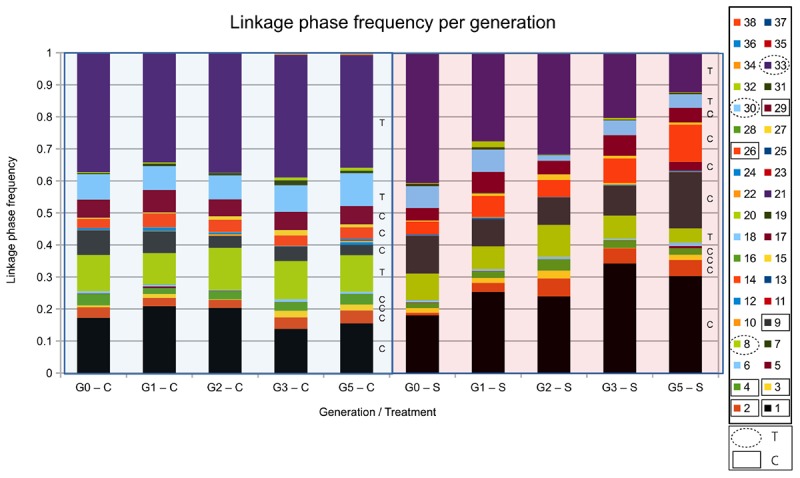
Linkage phase disequilibria during the selection process. Frequency of the haplotypes calculated in PHASE for each generation of selection. The first letter in the *x*-axis label indicates generation of selection (G0, G1,…, G5) and the last letter indicates treatment [Control (C) or Selection (S)]. Linkage phase disequilibrium was calculated for the six SNPs that were located in the highly supported region of selection as indicated by Jalvingh et al. (2014). Allelic composition and change in frequency of the eight haplotyptes that showed a frequency change larger than 0.01 are shown in Table 4. Many of the haplotypes were rare (<1%); for haplotypes that were more common (>2% in the fifth generation), we indicate whether it carried the C- or the T-allele for the gene *mbl*.

We calculated linkage phase disequilibrium between six SNP positions in the 600 kb genomic region on chromosome 2R, including *mbl* and *CG17287*, in PHASE (Stephens et al., 2001; Stephens and Donnelly, 2003) for each generation of selection in both the Control and Selection treatments (Figure 5). In the Control lines, the frequency of all combinations of alleles (haplotypes) remained very similar across the five generations of selection. In the Selection lines, five combinations of alleles increased in frequency (Haplotype 1, 2, 9, 17, and 26), and three allele combinations decreased in frequency throughout the selection process (Haplotype 8, 30, 33). Those haplotypes that increased in frequency consistently carried the C allele in *mbl*, while those that decreased in frequency consistently carried the T allele (Table 4). This supports the suggestion that the C-allele in the intron of *mbl* is a causal SNP that affects parasitoid resistance, or is closely linked to a causal variant that was favored by selection. In contrast, the G allele in *CG17287* that occurred in somewhat higher frequency in the Selection lines was present in haplotypes that decreased in frequency, while all other haplotypes that increased or decreased in frequency over time carried the T allele (Table 4).

**Table 4:** Allelic composition and frequency change of linkage phase disequilibria (“haplotypes”) that changed in frequency during the selection process for the S-lines and C-lines.

Haplotype	Direction	SNP in candidate gene						Frequency difference	
		*ark*	*RhoGEF2*	*CG17287*	*mbl*	*CG4844*	*CG42649*	S-lines	C-lines
8	-	C	T	**T**	**T**	C	A	-0.038	-0.001
30	-	G	G	**T**	**T**	G	G	-0.025	0
33	-	G	G	**T**	**T**	C	A	-0.285	0
									
1	+	C	T	**T**	**C**	G	G	0.123	0
2	+	C	T	**T**	**C**	G	A	0.04	-0.01
9	+	C	T	**G**	**C**	G	G	0.058	-0.005
17	+	C	G	**T**	**C**	C	A	0.027	0
26	+	G	G	**T**	**C**	G	G	0.076	0

*Linkage phase disequilibrium was calculated for the six SNPs that were located in the highly supported region of selection as indicated by Jalvingh et al. (2014). From left to right, these genes are: *ark, RhoGEF2, CG17287, mbl, CG4844* and *CG42649*. The genes that were significantly associated with parasitoid resistance (*CG17287* and *mbl*) are indicated by in bold. Linkage phase frequency was calculated in PHASE 2.1.1 and frequency differences were calculated based on the frequency before (generation 0) and after selection (generation 5) for the Selection and Control lines separately. Haplotype numbers correspond with those in Figure 5.*

## Discussion

In this study, we follow up on a previous “selection and re-sequence” study, where we selected *D. melanogaster* larvae for increased parasitoid resistance and identified SNP positions in the selected populations that had significantly changed in allele frequency. In the current study, we aimed to associate a subset of these SNPs to individual variation in resistance, and assess whether the same SNPs are associated with high resistance across different genetic backgrounds. Of the 94 candidate SNPs that changed significantly in frequency within a 600 kb region in the genome study (Jalvingh et al., 2014), we sequenced a subset of seven of these loci in Field lines, and found diverse patterns of genetic variation, including indications that selection may have acted on several of these SNPs. We selected 13 SNPs that we considered most likely to be the targets of selection, based on the genomic location of the SNP, signatures of selection of these SNPs in the Field lines that differ in parasitoid resistance, and the functional annotations of the gene in which they were present. We assessed for these SNPs whether they were significantly associated to the individual level of resistance. For three of the SNP positions we showed that the genotype was significantly associated to the individual variation in parasitoid resistance in the Selection and Control lines. This affirms that genetic variation in candidate genes identified by genome sequencing of populations can be linked with parasitoid resistance at the level of the individual. Furthermore, distinguishing which polymorphic positions are linked to individual resistance is an important first step toward understanding what the genetic basis is of increased parasitoid resistance in the Selection lines. For two of these SNPs, we also showed a consistent increase in allele frequency over the five generations of selection. However, we could not associate any of these SNPs to variation in parasitoid resistance in the 400 individually phenotyped individuals from 12 Field lines. Moreover, we found an indication that the effect of genotype for two of the SNPs was different across the Field lines. These results lead to the conclusion that alleles that might confer higher parasitoid resistance after experimental evolution may not be similarly associated to resistance in different genetic backgrounds or in natural populations that evolve more gradually under a variety of selective forces.

The current study was to disentangle the allelic variants in the rather large linkage block of 600 kb that we found in our earlier “evolve and re-sequence” study (Jalvingh et al., 2014). The repeatability of finding changes in genetic variation across the same linkage block for four Selection lines suggests the action of selection, not drift, on these positions. However, we expect within this region only a single (or few) SNP(s) to be the target of selection, while the other positions are swept along through linkage and genetic hitchhiking. As a first step to distinguish between the target(s) of selection and the SNPs that were swept along, we compared the presence and patterns of genetic variation for a set of seven candidate loci in eight Field lines of *D. melanogaster*. We analyzed the genetic variation across the Field lines, based on sequencing ∼500 bp segments of these seven candidate genes. The patterns of the genetic variation among natural population varied for the SNPs, with most showing a pattern that is consistent with neutral evolution. Examining in detail the differences, or the patterns of genetic variations, among the individual Field lines was not possible, as the small sample sizes per line would lead to under-sampling of the variation and unrepresentative population estimates (Goodall-Copestake et al., 2012). Across the Field lines, however, the seven candidate loci showed moderately high rates of genetic variation, with overall diversity indices that were similar to what was found for immunity genes by Obbard et al. (2009). A few SNPs showed patterns of diversity or higher divergence that were inconsistent with neutrality, and among those was a significant enrichment of the SNPs that had also significantly changed in frequency during experimental evolution. Thus, SNPs that were affected by experimental evolution for increased resistance were also those that show signs of selection in natural populations. When we associated individual genotype to phenotype measurements, however, none of these SNPs was consistently associated to parasitoid resistance in the Field lines.

In the Selection lines, we did find 3 SNPs that showed a statistical association with resistance at the individual level. In this individual-level analysis, we could distinguish more clearly which alleles were consistently associated to high resistance and which did not. The latter may have changed in frequency during experimental evolution due to chance association. Nonetheless, we cannot assign causality to these positions that we identified here as putative targets of selection, because we cannot exclude the possibility that a different causal variant segregated with these SNPs through genetic linkage, nor have we validated these three SNPs with functional characterization studies.

One SNP that was significantly associated with parasitoid resistance was located in the first intron of the gene *Musleblind* (*mbl*). Intronic polymorphisms can affect the regulation of gene expression and their splicing. *Mbl* is a large gene with many exons and many known splice variants (Pascual et al., 2006). It is associated with several biological processes, including photoreceptor cell differentiation, embryo development, muscle cell cellular homeostasis, regulation of female receptivity and regulation of gene expression (Flybase version FB2015_01, dos Santos et al., 2014). To the best of our knowledge, *mbl* has not been annotated with a role in defense or immunity, but this may need to be investigated. However, *Mbl* is associated to the *Ras* pathway, which is important in immunity and regulates cell proliferation and differentiation in multicellular eukaryotes, and overexpression of this pathway in *Drosophila* hemocytes results in overproliferation (Asha et al., 2003). Moreover, several recent publications reveal that muscles may play a role in regulating the immune response to parasitoids and hemocyte proliferation through the Jak/Stat pathway (Yang et al., 2015; Yang and Hultmark, 2017).

In our previous study, the gene *mbl* was associated with 24 segregating sites, all located in an intron of the gene, that showed a highly significant change in allele frequency (FDR adjusted *P* < 0.01). In the current study, we tested one of these SNPs. The SNP showed both a significant association of resistance level for one of the alleles in the Control and Selection lines, and a corresponding change of allele frequency during the selection process. In *mbl* the minor C-allele increased in frequency from approximately 40% in the founder population to 80% after five generations of selection. The observation that the selection process increased more than one haplotype may reflect the presence of the causal variant in multiple allelic backgrounds in the founder population. Since we selected from standing variation of a large outbred founder population, this was to be expected. Within the haplotype, the minor allele for the SNP in *mbl* was consistently increased in frequency over successive generations by selection, supporting the association of this SNP with individual immune ability, i.e., being a causal allele or closely linked to the causal variant (Table 4).

A SNP located in the non-coding part of exon 1 of the gene *methuselah-like 4* (*mthl4*), which was the (genotyped) SNP adjacent to *mbl* (see Figure 1), was also associated with resistance in the Selection line, and it steadily increased in frequency during the five generations of selection. In *mthl4* the minor A-allele increased in frequency from approximately 20% in the founder population to 50% after five generations of selection. The two haplotypes that were most prevalent and increased most strongly in the Selection lines shared the C-allele for *mbl* and the A-allele for *mthl4*. This SNP in *mthl4* also had the highest inter-population divergence, compared to neutral expectations, suggestive of being under positive selection in the Field lines. *Mthl4* showed the highest genetic diversity among the seven candidate loci that were sequenced in the Field lines, with the highest synonymous sites diversity among the tested genes, and the highest haplotype diversity (see Supplementary Table S5). Little information is available on this gene, except that it codes for a G-protein coupled receptor, and belongs to a gene family involved in the modulation of life span and stress responses (Brody and Cravchik, 2000). Several members of this gene family, including *mthl4* showed changed levels of gene expression in Selection lines for increased parasitoid resistance (Wertheim et al., 2011), while another member of the gene family showed upregulated expression from 12 h after parasitoid attack (Wertheim et al., 2005).

A SNP located in *CG17287*, also directly adjacent to *mbl* (see Figure 1), was also associated to resistance level and changed in allele frequency during selection, although primarily in the last generation and only is two of the four Selection lines. The SNP in *CG17287* is located in an exon but does not result in an amino acid change. The function of *CG17287* is not well known. It has zinc finger, DHHC-type and palmitoyltransferase protein motifs and may be associated to the endoplasmatic reticulum (Flybase version FB2015_01, dos Santos et al., 2014). The G-allele was rare in the Control lines (7%) and increased in frequency to ∼20% in the Selection lines. In the individual genotype–phenotype associations, the GG genotype was absent among the Control line sample, and only six individuals in the Selection line had this genotype, but all these individuals were resistant.

As mentioned before, to assess what, if any, role *mbl, mthl4* and *CH17287* genes have in parasitoid resistance, we would need to perform functional characterization. However, neither of these SNPs results in an amino acid change. Thus, assessing the expression, splicing and molecular function of these genes in resistant and susceptible larvae and populations will be needed to address which changes in gene function would have been caused by selection. Previous research showed that *mthl4* was differentially expressed in a Selection line for increased parasitoid resistance, showing a higher level of expression during early larval development (Wertheim et al., 2005). However, neither Wertheim et al. (2005, 2011) nor Salazar-Jaramillo et al. (2017) found evidence that either *mbl* or *CG17287* were significantly differently expressed after parasitization or after selection for increased parasitoid resistance. Possibly, the many SNPs in the intron of *mbl* could potentially affect the splicing of the transcripts of this gene, even through cryptic splicing sites, which could then result in trans-regulatory effects on other genes. One suggestion, therefore, would be to screen the mRNA of this gene for size-variations, and test for associations of mRNA length and resistance level. Alternatively, the genotype–phenotype associations that we found for the three adjacent SNPs are a strong indication that the allelic variant that was the target of selection is located somewhere within this haplotype unit among the three most central SNPs in the 600 kb region.

The genetic architecture of immunity is complex, with many genes involved. We tried to associate similar genotypes of individuals from different populations of *D. melanogaster* to parasitoid resistance. Consistent genotype–phenotype association patterns among lines are only to be expected when the same allele exists and would function in a similar way, i.e., confer a higher parasitoid resistance, in all lines. That was the implicit assumption for our study on Field lines, trying to untangle a linkage block that arose in a selection experiment for increased parasitoid resistance. If LD is present in narrower haplotype blocks in the Field lines, we could zoom in onto the region that is most closely linked to high parasitoid resistance. A lack of associations in the Field lines could arise when LD had entirely disappeared in the Field lines, so that the SNP markers are no longer linked to any causal variant, and thus non-informative for detecting a haplotype block that has a phenotypic effect on parasitoid resistance. We did find significant LD in the combined Field lines, and over narrower ranges than in the Control and Selection lines, which suggests that finding consistent genotype–phenotype associations across the 12 Field lines would have been feasible. However, for each individual Field line, LD was much higher and we found considerable differences in haplotype frequencies, indicative of the considerable population structuring that was also shown from microsatellite markers (Supplementary Table S3). Therefore, we cannot entirely exclude the possibility that, while LD exists between the adjacent SNP markers, it may show variable and inconsistent associations with any causal variant among the 12 Field lines. Yet, an alternative hypothesis is that allelic variation for parasitoid resistance is highly context-dependent: The influence of any particular allele may be strongly dependent on the genetic background and environmental factors that have been shaping the evolution of resistance levels in the different lines. Unfortunately, our analyses do not allow us to firmly distinguish the likelihood of any of these alternative explanations. We therefore recommend to compare the genotypes of resistant and susceptible individuals within a population and to associate their phenotypic variation in resistance to genetic variation in a genome-wide association study (GWAS). If we could do this for various natural populations, we may be able to identify the various evolutionary trajectories that may have led to the huge natural variation that we observe in resistance against parasitoids.

In our study, we used two different parasitoid strains to assess resistance. When specificity in host–parasitoid interactions is high, this could also provide yet another explanation for the difference between the Field lines and the Selection lines in the associations between resistance and specific SNPs. However, extensive research has shown that the resistance of *D. melanogaster* against *A. tabida* does not reflect genotype-specific co-evolution among pairs of host and parasitoid populations. The virulence mechanism of *A. tabida* consists of evading the immune response by adhering of the parasitoid eggs to host tissue, which prevents encapsulation by the hosts’ hemocytes (Kraaijeveld and van Alphen, 1994; Monconduit and Prévost, 1994; Eslin et al., 1996). There is a cline in virulence for *A. tabida* from the north (low) to the south (high) in Europe, and a corresponding cline in resistance in the hosts. Yet, the geographic patterns of parasitoid virulence and host resistance show no specific coupling of pairs of host and parasitoid populations (Kraaijeveld and van Alphen, 1994; Kraaijeveld and Godfray, 1999). Instead, southern parasitoid populations have more “sticky” eggs, and these more virulent parasitoid strains are more successful against all host strains, not specifically against their sympatric host strain. The outcome of 20 sympatric parasitoid–host associations could be largely predicted by the virulence and resistance of each strain against a reference strain (Kraaijeveld and Godfray, 2001). Moreover, artificial selection for increased defensive ability of the host raised resistance against a variety of different parasitoid strains that were tested, not specifically against the host strain that was used during artificial selection (Kraaijeveld and Godfray, 1999; Wertheim et al., 2011). Finally, we tested parasitoid resistance of eight of our Field lines against both parasitoid strains (Gerritsma et al., 2013), and found a strong correlation in the encapsulation rates. Thus, we consider it highly unlikely that the use of two parasitoid strains caused our finding of genotype–phenotype associations for some SNPs in the Selection lines, but not in the Field lines. We conclude that it more likely reflects either that different SNPs may have been selected for higher resistance in different genetic backgrounds and populations, or that the candidate SNPs we have identified in the Selection lines are not causal but linked to causal SNPs in the Selection lines, while not being consistently associated with these same SNPs in the Field lines.

## Conclusion

In conclusion, we studied the genetic basis of the variation in immune response against parasitoids among Field lines of *D. melanogaster* and in lines selected for parasitoid resistance. We aimed to find actual targets of selection for parasitoid resistance within a 600 kb block on chromosome 2R that showed signatures of selection in a whole-genome comparison of Control and Selection lines for parasitoid resistance (Jalvingh et al., 2014). Although we were successful in finding diverse patterns of the genetic variation among natural population in seven candidate genes within this 600 kb block, we did not obtain concrete evidence for any of our sequenced genes being candidates for parasitoid resistance across the Field lines. Our SNP genotyping assay failed to show any consistent associations between genotypes and level of resistance for the Field lines, while in the Selection and Control lines, three SNPs in *mbl, mthl4* and *CG17287*, were linked to variation in parasitoid resistance. This could imply that we selected the wrong candidate genes, and that another SNP was the actual target of selection for increased parasitoid resistance. The causal SNP is then most strongly linked to the three most central SNPs that we assessed in the 600 kb region. Alternatively, a lack of consistent associations in the Field lines was perhaps to be expected in the context of dynamic evolutionary landscapes. For a complex trait that evolves both fast and under local and spatially heterogeneous selection pressures, we may have to assume that the genetic basis of evolutionary adaptations is unlikely to converge among natural populations. Instead, we propose that within-line co-adapted gene complexes, consisting of different allelic combinations in different field lines, may be of more importance for determining resistance than any particular allele.

## Ethics Statement

All experiments on the insects have been performed in accordance with the relevant institutional and national guidelines and regulations. The study on these insects is exempted from ethics approval, and no genetically modified organisms were used.

## Author Contributions

SG, KJ, and BW contributed to the conception and design of the study. SG, KJ, CvdB, JB, and BW acquired and analyzed the data. KV coordinated and performed the SNP genotyping. SG and KJ wrote the first draft of the manuscript. SG, KJ, LvdZ, and BW wrote sections of the manuscript. All authors contributed to manuscript revision, read and approved the submitted version.

## Conflict of Interest Statement

The authors declare that the research was conducted in the absence of any commercial or financial relationships that could be construed as a potential conflict of interest.

## Footnotes

1 www.codoncode.com
