# Supplementary material for: Natural and Artificial Selection for Parasitoid Resistance in Drosophila melanogaster Leave Different Genetic Signatures
Source: Front Genet. 2019 May 31;10:479. doi: 10.3389/fgene.2019.00479 (PMC6557190; doi:10.3389/fgene.2019.00479)
Supplement: Supplementary file 1 [file Table_1.docx]

Supplementary Material

Natural and artificial selection for parasitoid resistance in *Drosophila melanogaster* leave different genetic signatures

**Sylvia Gerritsma^#^, Kirsten M. Jalvingh^#^, Carmen van de Beld, Jelmer Beerda, Louis van de Zande, Klaas Vrieling, Bregje Wertheim^*^**

*** Correspondence:** Corresponding Bregje Wertheim: b.wertheim@rug.nl

# Supplementary Material and Methods (for Supplementary Tables S1-S3)

## Microsatellite analysis

We conducted a microsatellite marker study to determine the population structure of the Field lines. Sixteen microsatellite markers were used in four multiplex PCR sets (MP1 –MP4). Chromosome location, primer sequences, fluorescent dye color of primer end-labels and repeat motifs are shown in Supplementary Table S1. Allele size range (bp) and number of alleles are based on the tested 8 *D. melanogaster* field lines used in this analysis, and summarized in Supplementary Table S2. The analysis of genetic differentiation among the 8 Field lines is presented in Supplementary Table S3.

**DNA isolation** DNA was extracted from 12 virgin females per field line using a high-throughput DNA extraction method adjusted after Hoarau et al., (2007). Tissue was homogenized in 50 µl digestion buffer (100 mM NaCl, 10 mM Tris-HCl pH 8.0, 25 mM EDTA pH 8.0, 0.5% SDS ) using tip-melted filter tips. After homogenizing, 50 µl digestion buffer containing 2 µl of 0.4 mg/ml proteinase K was added and mixed well. The samples were incubated overnight at 55°C, after which 40 µl of 6M NaCl and 100 ul Chloroform was added to each sample and mixed well. Samples were centrifuged for 20 min at 3000 rpm. The supernatant was transferred to a Millipore (MSFBN6B50) filter plate that contained an equal volume of binding buffer (0.908 g/ml NaI, 15 mg/ml Na_2_SO_3,_ dissolved in milliQ-filtered water ("MQ" from here on onwards)). The filter plate was centrifuged for 15 minutes at 1000 rpm and then 10 minutes at 2000 rpm to remove waste products (collected in waste collection plate). An equal volume to the supernatant of ice-cold wash buffer (freshly prepared solution of 100% EtOH and stock wash buffer: 100 mM NaCl, 20 mM Tris-HCL pH 8.0, 1 mM EDTA pH 8.0), was added to each sample, and centrifuged again for 10 minutes at 3000 rpm. This washing step was repeated three times due to the high salt concentrations in the samples. After discarding the waste, the plate was left to dry at RT for 30 minutes. DNA was eluted with 100 µl warm (55 °C) elution buffer (1M Tris-HCL pH 8.0, 0.1M EDTA) and incubated for 5 minutes. Plates were centrifuged for 5 minutes at 1000 rpm, followed by 5 minutes at 2000 rpm to collect the DNA in the PCR plates. DNA was diluted 10 times to make work solutions for multiplex PCR.

**Multiplex PCR** Sixteen microsatellite markers were amplified using the primers listed in Supplementary Table S1. All primer combinations were tested individually for use in a multiplex PCR. Products did not exceed 400 bp and were non-overlapping. Forward primers were end-labeled with either Hex or Fam (Biolegio, Nijmegen) fluorescent dye for scoring of the markers. With the available primers, four multiplex reactions could be made. Per reaction, 2.5 µl Qiagen® multiplex PCR master mix (HotStarTaq® DNA polymerase, Multiplex PCR buffer containing 6 mM MgCl_2_, dNTP mix) was added to 0.5 µl of the multiplex primer mix and 1 µl of the DNA sample. 1 µl of MQ was added to get a total reaction volume of 5 µl. PCR reaction was as follows: denaturation at 94 °C for 15 min, 30 cycles of 94 °C for 30 sec, 57 °C for 1½ min, 72 °C for 60 sec, then final extension at 72 °C for 45 min. PCR products were diluted 40 times with MQ (5 µl PCR product plus 195 µl MQ). 1.5 µl of the product was then added to 13.5 µl EDTA with size standard (4.5 µl size standard (Applera, the Netherlands) diluted into 1495.5 µl 0.2 mM EDTA), denatured for 2 minutes at 96 °C and put on ice and analyzed on an ABI 3730 automatic DNA sequencer.

**Analysis** Alleles were scored twice, independently, using Genemapper 4.0. Allelic richness was calculated using FSTAT 2.9.3. (Goudet, 1995). Number of alleles, number of private alleles and heterozygosity (H_o_) were evaluated using GenAlEx version 6.501 (Peakall and Smouse, 2006, 2012). Estimations of genetic differentiation (F_ST_) were evaluated using GenAlEx version 6.501 (Peakall and Smouse, 2006, 2012).

# References

Goudet, J. (1995). FSTAT (Version 1.2): A computer program to calculate F-statistics. *J. Hered.* 86, 485–486.

Hoarau, G., Coyer, J. a., Stam, W. T., and Olsen, J. L. (2007). A fast and inexpensive DNA extraction/purification protocol for brown macroalgae. *Mol. Ecol. Notes* 7, 191–193. doi:10.1111/j.1471-8286.2006.01587.x.

Jalvingh, K. M., Chang, P. L., Nuzhdin, S. V, and Wertheim, B. (2014). Genomic changes under rapid evolution: selection for parasitoid resistance. *Proc. R. Soc. B Biol. Sci.* 281, 20132303. doi:10.1098/rspb.2013.2303.

Peakall, R., and Smouse, P. E. (2006). GENALEX 6: genetic analysis in Excel. Population genetic software for teaching and research. *Mol. Ecol. Notes* 6, 288–295.

Peakall, R., and Smouse, P. E. (2012). GenAlEx 6.5: genetic analysis in Excel. Population genetic software for teaching and research-an update. *Bioinformatics* 28, 2537–2539.

# Supplementary Tables

## Supplementary Table S1

Summary of the microsatellite markers for the analysis of population structure in *D. melanogaster* Field lines. Sixteen microsatellite markers were used in four multiplex PCR sets (MP1 - MP4). Chromosome location, primer sequence, fluorescent dye colour of primer end-labels and repeat motifs are shown. Allele size range (bp) and number of alleles are based on the tested 96 individuals from the *D. melanogaster* Field lines used in this analysis (12 individuals per line, 8 lines). Genetic location (cM), cytological location and Genbank accession numbers are given.

|  | Name | Chr | Forward primer | Reverse primer | Color label dye | Repeat motif | Allele size range (bp) | Number of alleles | Genetic location (cM) | Cytological location | Genbank Acc.# |
| --- | --- | --- | --- | --- | --- | --- | --- | --- | --- | --- | --- |
| MP1 | DM21 | 2L | TAATGGGGAATGGGTGAATG | GCCGTGCTCTTTTCTCTTACG | fam | (TG)19 | 81-131 | 18 | 4 | 22C | M97694 |
|  | X9928573gt(X9) | X | GTTGTGCCTCTGCCAGTCAGTC | GAATTATTTCACGATTATCTTCAGG | hex | (GT)13 | 133-145 | 6 | 1-30 | 9B3 | - |
|  | DM3G | 3L | TCCTCCTGCTCAACCATTTC | TTTAACGATGTCTTGGCGAC | hex | (GT)13 | 167-187 | 8 | 3-15 | 64D | - |
|  | DMU12269 | 2L | TGGGATCCGTGGATCATAGT | ATTCGGGAATGAGGACAGTG | fam | (AAC)7 | 231-252 | 7 | 39 | 31A1-A3 | DMU12269 |
|  | DM30 | 3L | TATCCTATGCAAACACAGGCC | GGCCATAACTGAAAAGCTATGC | hex | (ACC)5 | 369-378 | 4 | 1.5 | 62B4-5 | L32839 |
| MP2 | DM22 | 2R | ACAGCAACAACGGAGCAAC | TCTGCAACCTGGGAGTCTG | fam | (CAG)7 | 73-79 | 3 | 8-87 | 54F | X15657 |
|  | Tor | 2R | TGCAGTCATCAATGGCTAATC | TGATTTCCCCCGTCCGAAGTG | hex | (CA)13 | 98-112 | 7 | 56 | 43B3-C5 | - |
|  | DROGPAD | 2R | GAAATAGGAATCATTTTGAATGGC | AATTAAAAACAAAAAACCTGAGCG | fam | (GT)19 | 171-195 | 6 | 60 | 47A | M31129 |
|  | DMPROSPER | 3R | CGGTACAAAGTGTGTGTTC | GACTTTTAAACATTTAAGATTAATTCC | hex | (GA)12 | 195-209 | 6 | 50 | 8.60E+02 | Z11743 |
|  | DMU566661 | X | TATTTCGCTAACAAACCGGC | AACGCGATCACAAACATCAA | hex | (AC)15 | 250-282 | 11 | 1-9 | 4F1-F2 | U566661 |
| MP3 | DM28 | 2R | AGCCACAGCCATGCGTTTAAC | CACACGCTGACAGGATCTACT | hex | (GT)8 | 93-121 | 9 | 101 | 59a1-b2 | - |
|  | AC004373 | 2L | AATGCGTGTGTTTGGATGAA | GTCCCAGTCTCCCAGTGAAA | fam | (AT)15 | 179-191 | 6 | 2-12.5 | 24F1-F2 | AC004307 |
|  | DMC114E2 | X | CAACTGCAGCAGCAACAAAT | ATTCGTAAGTTGCCCGTCTG | hex | (AT)17 | 318-324 | 4 | 3.5 | 3D-E | Z98254 |
| MP4 | DMX2 | X | CAAGAGATCCCGAGAGAGAGA | ACGTGTGCGTGTTGTTTCTC | hex | (CA)11 | 79-93 | 4 | 57.6 | 16F3-6 | X58188 |
|  | DM24 | 2L | CATTGGAAAAGTGAGCGGAT | CGGACAACAACAAATCGTTG | fam | (CT)7 | 131-133 | 2 | 17.8 | 25F5-26A | J04567 |
|  | AF221066 | 3R | GCCGACAATTACTGGCATTT | CTTTGCGTCTGTTCAATTGTG | hex | (TA)16 | 186-226 | 13 | 3-103 | 100F5 | AF221066 |

## Supplementary Table S2

Summary of genetic parameters based on the microsatellite analysis using 16 markers (described in Supplementary Table S1) and 8 Field lines. Sample size (N) refers to the number of individuals used for the microsatellite analyses, total number of observed alleles (No. of alleles), allelic richness (AR, averaged over all markers), number of private alleles (PA), observed (H_o_) and expected (H_e_) heterozygosity and the fixation index per population with corresponding standard error are shown.

Private alleles were found in all 8 lines, with percentage of private alleles ranging from 3.1% to 18.8%, which suggests considerable differentiation among the Field lines. Heterozygosity levels were lowest for STA, 0.295 ± 0.060. All other lines showed observed heterozygosity levels ranging from 0.417 to 0.540, and did not deviate from expected heterozygosity values (mean observed heterozygosity for all the lines was 0.445 ± 0.025 and mean expected heterozygosity 0.467 ± 0.021), indicating that the Field lines are genetically variable at a similar level. The overall fixation index F was 0.0625 ± 0.025, which suggest random mating within the field lines. STA showed a relatively high fixation index, compared to the other lines, namely 0.217 ± 0.070, which could indicate inbreeding or undetected null alleles.

| Line | Location | N | No. of alleles | AR | | | PA | Ho | | | He | | | F | | |
| --- | --- | --- | --- | --- | --- | --- | --- | --- | --- | --- | --- | --- | --- | --- | --- | --- |
| ARL | Arles, France | 12 | 50 | 3.008 | ± | 0.336 | 1 | 0.417 | ± | 0.073 | 0.466 | ± | 0.063 | 0.123 | ± | 0.072 |
| BAY | Bayreuth, Germany | 12 | 58 | 3.470 | ± | 0.305 | 5 | 0.505 | ± | 0.058 | 0.500 | ± | 0.052 | -0.014 | ± | 0.054 |
| BRE | Bremen, Germany | 12 | 59 | 3.443 | ± | 0.319 | 6 | 0.429 | ± | 0.062 | 0.457 | ± | 0.056 | 0.033 | ± | 0.068 |
| GOT | Gotheron, France | 12 | 58 | 3.306 | ± | 0.305 | 6 | 0.448 | ± | 0.073 | 0.431 | ± | 0.064 | -0.009 | ± | 0.057 |
| GRO | Groningen, Netherlands | 12 | 56 | 3.340 | ± | 0.347 | 1 | 0.474 | ± | 0.087 | 0.489 | ± | 0.064 | 0.105 | ± | 0.097 |
| INN | Inssbruck, Austria | 12 | 67 | 3.912 | ± | 0.380 | 5 | 0.540 | ± | 0.069 | 0.533 | ± | 0.059 | 0.001 | ± | 0.061 |
| KAL | Kaltern am See, Italy | 12 | 59 | 3.478 | ± | 0.346 | 6 | 0.455 | ± | 0.074 | 0.489 | ± | 0.061 | 0.102 | ± | 0.081 |
| STA | St Andrews, Scotland | 12 | 49 | 2.887 | ± | 0.420 | 2 | 0.295 | ± | 0.060 | 0.375 | ± | 0.061 | 0.217 | ± | 0.070 |
| Total |  | 96 | 456 |  |  |  | 32 |  |  |  |  |  |  |  |  |  |
| Mean |  |  | 57 | 3,355 | ± | 0.122 | 4 | 0.445 | ± | 0.025 | 0.467 | ± | 0.021 | 0.065 | ± | 0.025 |

## Supplementary Table S3

Pairwise F_ST_ values (above diagonal) and pairwise G_ST_ values (below diagonal) based on microsatellite analysis of the *D. melanogaster* Field lines. The probability of a random value greater than or equal to the observed value was tested based on 999 permutations of the data (*P*<0.05). All pairwise comparisons were significant, indicating genetic differentiation among all lines (F_ST_=0.148±0.014; 95% CI 0.125-0.181, G_st_=0.111±0.014; 95% CI 0.089-0.127). The F_ST_ values ranged from 0.037 to 0.168, with STA being the line that was most genetically differentiated to all other lines.

| Line | Location | Pairwise F_ST_ (Wright) | | | | | | | |
| --- | --- | --- | --- | --- | --- | --- | --- | --- | --- |
|  |  | BAY | STA | GRO | BRE | INN | KAL | ARL | GOT |
| BAY | Bayreuth, Germany | - | 0.125 | 0.061 | 0.097 | 0.051 | 0.068 | 0.100 | 0.085 |
| STA | St Andrews, Scotland | 0.101 | - | 0.133 | 0.168 | 0.087 | 0.112 | 0.146 | 0.148 |
| GRO | Groningen, Netherlands | 0.038 | 0.108 | - | 0.106 | 0.044 | 0.055 | 0.061 | 0.070 |
| BRE | Bremen, Germany | 0.075 | 0.143 | 0.082 | - | 0.108 | 0.112 | 0.121 | 0.104 |
| INN | Inssbruck, Austria | 0.029 | 0.063 | 0.022 | 0.085 | - | 0.037 | 0.065 | 0.061 |
| KAL | Kaltern am See, Italy | 0.045 | 0.087 | 0.032 | 0.088 | 0.014 | - | 0.090 | 0.071 |
| ARL | Arles, France | 0.077 | 0.120 | 0.037 | 0.097 | 0.042 | 0.066 | - | 0.051 |
| GOT | Gotheron, France | 0.063 | 0.124 | 0.047 | 0.081 | 0.039 | 0.048 | 0.028 | - |
|  |  | BAY | STA | GRO | BRE | INN | KAL | ARL | GOT |
|  |  | Pairwise G_ST_ (Nei) | | | | | | | |

## Supplementary Table S4

Summary of the candidate genes for the analysis of genetic variation in *D. melanogaster* Field lines. Flybase gene number (Fbgn), chromosome location (Chr), and sense (+) or antisense (-) DNA strand sequence are given. SNP ID and genomic position refer to the name and genomic location of the SNP found in Jalvingh et al., (2014). Type refers to the part of the sequence that is amplified and includes the SNP from Jalvingh et al (2014); Exon-NC stands for a non-coding part of the exon. Primer sequences, annealing temperature (T_a_) and amplicon length (bp) are shown.

| Gene | Fbgn | Chr | DNA strand | SNP ID | Genomic pos SNP | Type | Forward primer | Reverse primer | T_a_ | Amplicon (bp) |
| --- | --- | --- | --- | --- | --- | --- | --- | --- | --- | --- |
| Ark | FBgn0024252 | 2R | + | *Ark*_689_Jal | 12911689 | Exon | TAACCAACAGAAGCACTTGATCAC | CCGTTGGTCAAATCCCATACAG | 54 | 408 |
| RhoGEF2 | FBgn0023172 | 2R | + | *RhoGEF2*_133_Jal | 12930133 | Exon | TCTCTTACTGGAGTTCAGTTGCG | TGGCAATGTCCACAATCTGGT | 52 | 544 |
|  |  | 2R |  | *RhoGEF2*_160_Jal | 12930160 | Exon |  |  |  |  |
| CG6568 | FBgn0034210 | 2R | - | *CG6568*_750_Jal | 13293750 | Exon | CGATCAACGAGCGTACATGC | CAAGCTGTGTTCATCAAGAGG | 52 | 400 |
| Mthl4 | FBgn0034219 | 2R | - | *Mthl4*_812_Jal | 13334812 | Exon-NC | AAGGGATCGTGCTTGTTCAG | TTTGCGGAGGTTCTTTTGCTA | 53 | 475 |
|  |  | 2R |  | *Mthl4*_827_Jal | 13334827 | Exon-NC |  |  |  |  |
| CG11423 | FBgn0034251 | 2R | + | *CG11423*_886_Jal | 13483886 | Intron | TTGGACCACTTGCTGATGCT | CATACTGAAGATTGCAAGCTTCG | 52 | 419 |
| CG42649 | FBgn0261501 | 2R | - | *CG42649*_693_jal | 13486693 | Intron | TCCGTATGGATTAAAAGGTGGT | TCCACACGAACGTTTCAACA | 52 | 596 |
|  |  | 2R |  | *CG42649*_817_jal | 13486817 | Intron |  |  |  |  |
|  |  | 2R |  | *CG42649*_933_jal | 13486933 | Intron |  |  |  |  |
| CG17287 | FBgn0034202 | 2R | - | *CG17287*_201_Jal | 13045201 | Exon | TGAAGAATGTTCCAGGAATGC | TGACCATTGGACTGTTGCTC | 50 | 496 |
|  |  | 2R |  | *CG17287*_210_Jal | 13045210 | Exon |  |  |  |  |
|  |  | 2R |  | *CG17287*_219_Jal | 13045219 | Exon |  |  |  |  |
|  |  | 2R |  | *CG17287*_324_Jal | 13045324 | Exon |  |  |  |  |

## Supplementary Table S5

Pairwise *F_ST_* values for all field lines, based on 116 SNPs found in the candidate genes, are shown above diagonal. G_ST_ values could not be calculated due to missing values. The probability of a random value greater than or equal to the observed value was tested based on 999 permutations of the data (p<0.05). All pairwise comparisons were non-significant.

| Line | Location | Pairwise Fst (Wright) | | | | | | | |
| --- | --- | --- | --- | --- | --- | --- | --- | --- | --- |
|  |  | BAY | STA | GRO | BRE | INN | KAL | ARL | GOT |
| BAY | Bayreuth, Germany | - | 0.000 | 0.000 | 0.000 | 0.000 | 0.000 | 0.000 | 0.000 |
| STA | St Andrews, Scotland |  | - | 0.036 | 0.000 | 0.000 | 0.000 | 0.000 | 0.000 |
| GRO | Groningen, Netherlands |  |  | - | 0.034 | 0.024 | 0.027 | 0.054 | 0.000 |
| BRE | Bremen, Germany |  |  |  | - | 0.000 | 0.000 | 0.000 | 0.000 |
| INN | Inssbruck, Austria |  |  |  |  | - | 0.000 | 0.000 | 0.000 |
| KAL | Kaltern am See, Italy |  |  |  |  |  | - | 0.000 | 0.000 |
| ARL | Arles, France |  |  |  |  |  |  | - | 0.000 |
| GOT | Gotheron, France |  |  |  |  |  |  |  | - |
|  |  | BAY | STA | GRO | BRE | INN | KAL | ARL | GOT |
|  |  |  | | | | | | | |

## Supplementary Table S6

Summary of genetic parameters of sequenced fragments of 7 candidate genes, summarized per gene, and analysed for all Field lines pooled together. The numbers of SNPs are listed in the table, but not all SNPs could be included into the analyses due to gaps. These excluded SNPs are indicated by an asterisk (*) and are located in the genes *Ark*: 7 synonomous SNPs, *RhoGEF2*: 9 synonomous SNPs and *Mthl4*: 2 non-coding SNPs. Total number of analysed sequences (N__seq_); polymorphic sites categorized for synonymous (Syn), nonsynonymous (NonSyn) and non-coding; average synonymous nucleotide diversity (π_s_); amino acid diversity (π_a_); observed heterozygosity (H_o_); expected heterozygosity (H_e_); number of haplotypes (#h); haplotype diversity (hd); Tajima’s D for synonymous sites (D__Syn_) and nonsynonymous sites (D__NonSyn_).

|  |  |  | Polymorphic sites | | | Nucleotide diversity | | Heterozygosity | | | | | | Haplotypes | | Tajima's D | |
| --- | --- | --- | --- | --- | --- | --- | --- | --- | --- | --- | --- | --- | --- | --- | --- | --- | --- |
| Gene | Annotation | N_seq | Syn | NonSyn | Non-coding | πs | πa | Ho | | | He | | | #h | hd | D_Syn | D_NonSyn |
| *ark* | Apoptosis/cell death | 80 | 8* | 4 | - | 0.0290 | 0.0007 | 0.145 | ± | 0.027 | 0.157 | ± | 0.022 | 13 | 0.74 | 0.910 | -1.622 |
| *RhoGEF2* | Cell morphogenesis | 96 | 10* | 4 | - | 0.0123 | 0.0002 | 0.089 | ± | 0.017 | 0.085 | ± | 0.015 | 9 | 0.63 | -0.365 | **-1.783** |
| *CG6568* | - | 96 | 5 | 3 | - | 0.0259 | 0.0006 | 0.313 | ± | 0.041 | 0.231 | ± | 0.027 | 9 | 0.77 | **2.252** | -1.370 |
| *mthl4* | G-protein-coupled binding receptor/cell surface receptor, Involved in stress response | 70 | 12 | 11 | 9* | 0.0342 | 0.0036 | 0.151 | ± | 0.016 | 0.123 | ± | 0.011 | 27 | 0.94 | 0.014 | -1.679 |
| *CG11432* | Oxidative phosporylation pathway | 96 | - | - | 13 | 0.0371 (nc) | - | 0.228 | ± | 0.021 | 0.209 | ± | 0.018 | 11 | 0.72 | 0.730 | - |
| *CG42649* | - | 88 | - | - | 18 | 0.0065 (nc) | - | 0.112 | ± | 0.015 | 0.131 | ± | 0.015 | 22 | 0.87 | -0.885 | - |
| *CG17287* | Metal-binding | 186 | 13 | 6 | - | 0.0166 | 0.0011 | 0.109 | ± | 0.012 | 0.102 | ± | 0.011 | 15 | 0.56 | -0.701 | -1.274 |
| * Not all in DNAsp analyses, due to gaps | | |  |  |  |  |  |  |  |  |  |  |  |  |  |  |  |

## Supplementary Table S7

Genetic parameters of sequenced fragments of the candidate genes, summarized per gene per Field line. Total number of analyzed sequences (N__seq_), polymorphic sites categorized for synonymous (Syn), nonsynonymous (NonSyn) and non-coding positions, and total number of polymorphic sites (Total S) are shown. SNPs included in DNAsp analyses are shown between brackets. SNPs not included into the analyses are located in *Ark*: 7 synonomous SNPs, *RhoGEF2*: 9 synonomous SNPs and *Mthl4*: 2 non-coding SNPs. Nucleotide diversity (π) calculated over all synonymous and nonsynymous sites grouped together (π) and separately (respectively, π_s_, π_a_) is shown. When the gene fragment consists of non-coding DNA sequence, nucleotide diversity falls in the column π non-coding. Observed (H_o_) and expected (H_e_) heterozygosity, number of haplotypes (#h) and haplotype diversity (hd), and Tajima’s D for synonymous (D__Syn_) and nonsynonymous (D__NonSyn_) sites are shown. Summarized genetic parameters per gene are shown in bold. Significant values of Tajima’s D are shown in red and italic.

* One individual from BAY contained a nonsynonymous SNP (position 12911449 in *Ark*), which translated to a stop-codon.

** For STA in gene *CG42649*, sequences of one individual were excluded because it contained a 11 bp gap including 2 polymorphic sites, which would be treated as missing data in DNAsp otherwise. Heterozygosity was calculated using GenAlEx in Excel, all other parameters were calculated using DNAsp.

|  |  |  | **Polymorphic sites** | | | | **Nucleotide diversity (π)** | | | | **Heterozygosity** | | | | | | **Haplotypes** | | **Tajima's D** | |
| --- | --- | --- | --- | --- | --- | --- | --- | --- | --- | --- | --- | --- | --- | --- | --- | --- | --- | --- | --- | --- |
| **Gene** | **Line** | **N_ seq** | **Syn** | **Non-Syn** | **Non-coding** | **Total S** | **π** | **πs** | **πa** | **π non-coding** | **Ho** | | | **He** | | | **#h** | **hd** | **D_**  **Syn** | **D_**  **NonSyn** |
| ***ark*** | **Total** | **80** | **8 (7 in DNAsp)** | **4** | **-** | **12 (11 in DNAsp)** | **0.0070** | **0.0290** | **0.0007** | **-** | **0.145** | **±** | **0.027** | **0.157** | **±** | **0.022** | **13** | **0.74** | **0.910** | **-1.622** |
|  | STA | 12 | 3 | 0 | **-** | 3 | 0.0043 | 0.0165 | 0.0000 | **-** | 0.125 | **±** | 0.074 | 0.101 | **±** | 0.054 | 5 | 0.79 | 1.072 | - |
|  | BAY | 8 | 6 | 2* | **-** | 8 | 0.0101 | 0.0290 | 0.0031 | **-** | 0.069 | **±** | 0.037 | 0.247 | **±** | 0.046 | 5 | 0.79 | -0.201 | 0.414 |
|  | GRO | - | **-** | **-** | **-** | **-** | **-** | **-** | **-** | **-** | **-** |  | **-** | **-** | **-** | **-** | **-** | **-** | **-** | **-** |
|  | BRE | 12 | 4 | 0 | **-** | 4 | 0.0062 | 0.0211 | 0.0000 | **-** | 0.111 | **±** | 0.056 | 0.144 | **±** | 0.062 | 3 | 0.71 | 1.472 | - |
|  | INN | 12 | 4 | 1 | **-** | 5 | 0.0061 | 0.0191 | 0.0005 | **-** | 0.236 | **±** | 0.109 | 0.142 | **±** | 0.057 | 4 | 0.71 | 0.989 | -1.141 |
|  | KAL | 12 | 5 | 0 | **-** | 5 | 0.0056 | 0.0222 | 0.0000 | **-** | 0.167 | **±** | 0.065 | 0.130 | **±** | 0.049 | 5 | 0.67 | 0.092 | - |
|  | ARL | 12 | 4 | 0 | **-** | 4 | 0.0067 | 0.0228 | 0.0000 | **-** | 0.194 | **±** | 0.084 | 0.155 | **±** | 0.066 | 3 | 0.71 | 1.874 | - |
|  | GOTH | 12 | 4 | 1 | **-** | 5 | 0.0078 | 0.0239 | 0.0010 | **-** | 0.111 | **±** | 0.047 | 0.181 | **±** | 0.066 | 4 | 0.73 | 1.954 | -0.195 |
| ***RhoGEF2*** | **Total** | **96** | **10 (9 in DNAsp)** | **4** | **-** | **14 (13 in DNAsp)** | **0.0030** | **0.0123** | **0.0002** | **-** | **0.089** | **±** | **0.017** | **0.085** | **±** | **0.015** | **9** | **0.63** | **-0.365** | ***-1.783*** |
|  | STA | 12 | 1 | 0 | **-** | 1 | 0.0006 | 0.0024 | 0.0000 | **-** | 0.024 | **±** | 0.024 | 0.020 | **±** | 0.020 | 2 | 0.63 | -0.195 | - |
|  | BAY | 12 | 1 | 2 | **-** | 3 | 0.0017 | 0.0041 | 0.0008 | **-** | 0.036 | **±** | 0.019 | 0.057 | **±** | 0.036 | 3 | 0.59 | 1.381 | -1.451 |
|  | GRO | 12 | 1 | 1 | **-** | 2 | 0.0014 | 0.0041 | 0.0004 | **-** | 0.048 | **±** | 0.037 | 0.046 | **±** | 0.036 | 3 | 0.59 | 1.381 | -1.141 |
|  | BRE | 12 | 6 | 0 | **-** | 6 | 0.0046 | 0.0193 | 0.0000 | **-** | 0.214 | **±** | 0.075 | 0.163 | **±** | 0.056 | 4 | 0.82 | 0.956 | - |
|  | INN | 12 | 6 | 1 | **-** | 7 | 0.0029 | 0.0108 | 0.0004 | **-** | 0.119 | **±** | 0.048 | 0.097 | **±** | 0.034 | 3 | 0.53 | -1.283 | -1.141 |
|  | KAL | 12 | 5 | 0 | **-** | 5 | 0.0044 | 0.0174 | 0.0000 | **-** | 0.119 | **±** | 0.051 | 0.147 | **±** | 0.059 | 5 | 0.73 | 1.306 | - |
|  | ARL | 12 | 2 | 0 | **-** | 2 | 0.0012 | 0.0047 | 0.0000 | **-** | 0.048 | **±** | 0.032 | 0.040 | **±** | 0.027 | 2 | 0.30 | -0.248 | - |
|  | GOTH | 12 | 4 | 0 | **-** | 4 | 0.0033 | 0.0128 | 0.0000 | **-** | 0.107 | **±** | 0.062 | 0.108 | **±** | 0.049 | 4 | 0.71 | 0.868 | - |
| ***CG6568*** | **Total** | **96** | **5** | **3** | **-** | **8** | **0.0070** | **0.0259** | **0.0006** | **-** | **0.313** | **±** | **0.041** | **0.231** | **±** | **0.027** | **9** | **0.77** | ***2.252*** | **-1.370** |
|  | STA | 12 | 4 | 0 | **-** | 4 | 0.0044 | 0.0165 | 0.0000 | **-** | 0.229 | **±** | 0.104 | 0.165 | **±** | 0.067 | 3 | 0.62 | 0.305 | - |
|  | BAY | 12 | 4 | 0 | **-** | 4 | 0.0059 | 0.0239 | 0.0000 | **-** | 0.333 | **±** | 0.137 | 0.219 | **±** | 0.084 | 4 | 0.82 | 1.552 | - |
|  | GRO | 12 | 5 | 1 | **-** | 6 | 0.0072 | 0.0249 | 0.0006 | **-** | 0.375 | **±** | 0.103 | 0.267 | **±** | 0.067 | 5 | 0.85 | 1.138 | -1.141 |
|  | BRE | 12 | 4 | 1 | **-** | 5 | 0.0073 | 0.0251 | 0.0006 | **-** | 0.396 | **±** | 0.141 | 0.269 | **±** | 0.089 | 5 | 0.80 | *2.276* | -1.141 |
|  | INN | 12 | 4 | 1 | **-** | 5 | 0.0042 | 0.0125 | 0.0011 | **-** | 0.229 | **±** | 0.118 | 0.155 | **±** | 0.061 | 4 | 0.71 | -0.741 | -0.195 |
|  | KAL | 12 | 4 | 1 | **-** | 5 | 0.0066 | 0.0236 | 0.0006 | **-** | 0.375 | **±** | 0.129 | 0.247 | **±** | 0.081 | 4 | 0.74 | 1.753 | -1.141 |
|  | ARL | 12 | 4 | 1 | **-** | 5 | 0.0069 | 0.0241 | 0.0006 | **-** | 0.292 | **±** | 0.098 | 0.257 | **±** | 0.085 | 4 | 0.77 | *1.995* | -1.141 |
|  | GOTH | 12 | 4 | 1 | **-** | 5 | 0.0073 | 0.0251 | 0.0006 | **-** | 0.271 | **±** | 0.118 | 0.269 | **±** | 0.089 | 5 | 0.80 | *2.276* | -1.141 |
| ***mthl4*** | **Total** | **70** | **12** | **11** | **9 (2 in DNAsp)** | **32 (25 in DNAsp)** | **0.0090** | **0.0342** | **0.0036** | **-** | **0.151** | **±** | **0.016** | **0.123** | **±** | **0.011** | **27** | **0.94** | **0.014** | **-1.679** |
|  | STA | 7 | 4 | 1 | 2 | 7 | 0.0093 | 0.0277 | 0.0022 | **-** | 0.077 | **±** | 0.025 | 0.109 | **±** | 0.035 | 4 | 0.78 | 1.652 | 1.303 |
|  | BAY | 8 | 4 | 4 | 0 | 8 | 0.0075 | 0.0238 | 0.0048 | **-** | 0.109 | **±** | 0.030 | 0.113 | **±** | 0.030 | 6 | 0.93 | 0.586 | -1.030 |
|  | GRO | 7 | 4 | 3 | 0 | 7 | 0.0115 | 0.0365 | 0.0075 | **-** | 0.266 | **±** | 0.078 | 0.137 | **±** | 0.039 | 3 | 0.83 | 2.080 | 1.090 |
|  | BRE | 10 | 4 | 6 | 0 | 10 | 0.0055 | 0.0131 | 0.0049 | **-** | 0.081 | **±** | 0.020 | 0.072 | **±** | 0.017 | 7 | 0.87 | -1.245 | *-1.796* |
|  | INN | 9 | 5 | 2 | 1 | 9 | 0.0089 | 0.0326 | 0.0027 | **-** | 0.134 | **±** | 0.037 | 0.117 | **±** | 0.030 | 6 | 0.87 | 1.435 | -0.184 |
|  | KAL | 10 | 6 | 4 | 0 | 10 | 0.0082 | 0.0310 | 0.0039 | **-** | 0.169 | **±** | 0.038 | 0.142 | **±** | 0.032 | 8 | 0.93 | 0.284 | -1.245 |
|  | ARL | 12 | 7 | 2 | 0 | 9 | 0.0067 | 0.0313 | 0.0014 | **-** | 0.101 | **±** | 0.036 | 0.104 | **±** | 0.028 | 6 | 0.80 | -0.051 | -1.451 |
|  | GOTH | 12 | 8 | 2 | 2 | 12 | 0.0125 | 0.0483 | 0.0027 | **-** | 0.271 | **±** | 0.051 | 0.194 | **±** | 0.033 | 6 | 1.00 | 0.050 | -1.132 |
| ***CG11432*** | **Total** | **96** | **-** | **-** | **13** | **13** | **0.0099** | **0.0000** | **0.0000** | **0.0371** | **0.228** | **±** | **0.021** | **0.209** | **±** | **0.018** | **11** | **0.72** | **0.730** | **-** |
|  | STA | 12 | **-** | **-** | 3 | 3 | 0.0025 | **-** | **-** | 0.0378 | 0.077 | **±** | 0.041 | 0.064 | **±** | 0.034 | 2 | 0.30 | -0.278 | **-** |
|  | BAY | 12 | **-** | **-** | 7 | 7 | 0.0089 | **-** | **-** | 0.0347 | 0.154 | **±** | 0.044 | 0.233 | **±** | 0.065 | 4 | 0.80 | 1.663 | **-** |
|  | GRO | 12 | **-** | **-** | 10 | 10 | 0.0097 | **-** | **-** | 0.0284 | 0.205 | **±** | 0.038 | 0.252 | **±** | 0.054 | 5 | 0.82 | 0.328 | **-** |
|  | BRE | 12 | **-** | **-** | 10 | 10 | 0.0076 | **-** | **-** | 0.0295 | 0.269 | **±** | 0.077 | 0.198 | **±** | 0.047 | 5 | 0.73 | -0.632 | **-** |
|  | INN | 12 | **-** | **-** | 12 | 12 | 0.0140 | **-** | **-** | 0.0376 | 0.308 | **±** | 0.062 | 0.271 | **±** | 0.048 | 6 | 0.85 | -0.132 | **-** |
|  | KAL | 12 | **-** | **-** | 11 | 11 | 0.0089 | **-** | **-** | 0.0405 | 0.282 | **±** | 0.044 | 0.231 | **±** | 0.034 | 5 | 0.67 | -0.422 | **-** |
|  | ARL | 12 | **-** | **-** | 10 | 10 | 0.0094 | **-** | **-** | 0.0393 | 0.333 | **±** | 0.091 | 0.246 | **±** | 0.057 | 4 | 0.68 | 0.216 | **-** |
|  | GOTH | 12 | **-** | **-** | 9 | 9 | 0.0067 | **-** | **-** | 0.0284 | 0.192 | **±** | 0.046 | 0.174 | **±** | 0.042 | 5 | 0.58 | -0.695 | **-** |
| ***CG42649*** | **Total** | **88** | **-** | **-** | **18** | **18** | **0.0060** | **0.0000** | **0.0000** | **0.0065** | **0.112** | **±** | **0.015** | **0.131** | **±** | **0.015** | **22** | **0.87** | **-0.885** | **-** |
|  | STA** | 8 | **-** | **-** | 4 | 4 | 0.0040 | **-** | **-** | 0.0056 | 0.044 | **±** | 0.026 | 0.113 | **±** | 0.045 | 4 | 0.64 | 0.283 | **-** |
|  | BAY | 12 | **-** | **-** | 10 | 10 | 0.0079 | **-** | **-** | 0.0079 | 0.111 | **±** | 0.027 | 0.167 | **±** | 0.042 | 8 | 0.89 | -0.048 | **-** |
|  | GRO | 12 | **-** | **-** | 7 | 7 | 0.0058 | **-** | **-** | 0.0058 | 0.139 | **±** | 0.053 | 0.121 | **±** | 0.044 | 6 | 0.80 | 0.103 | **-** |
|  | BRE | 12 | **-** | **-** | 7 | 7 | 0.0048 | **-** | **-** | 0.0048 | 0.130 | **±** | 0.051 | 0.102 | **±** | 0.038 | 7 | 0.83 | -0.537 | **-** |
|  | INN | 10 | **-** | **-** | 7 | 7 | 0.0064 | **-** | **-** | 0.0064 | 0.044 | **±** | 0.020 | 0.133 | **±** | 0.042 | 6 | 0.89 | 0.329 | **-** |
|  | KAL | 12 | **-** | **-** | 12 | 12 | 0.0090 | **-** | **-** | 0.0090 | 0.157 | **±** | 0.037 | 0.191 | **±** | 0.043 | 8 | 0.92 | -0.553 | **-** |
|  | ARL | 10 | **-** | **-** | 6 | 6 | 0.0057 | **-** | **-** | 0.0057 | 0.156 | **±** | 0.064 | 0.118 | **±** | 0.045 | 6 | 0.87 | 0.458 | **-** |
|  | GOTH | 12 | **-** | **-** | 6 | 6 | 0.0048 | **-** | **-** | 0.0048 | 0.111 | **±** | 0.045 | 0.100 | **±** | 0.039 | 5 | 0.76 | -0.033 | **-** |
| ***CG17287*** | **Total** | **186** | **13** | **6** | **-** | **19** | **0.0050** | **0.0166** | **0.0011** | **-** | **0.109** | **±** | **0.012** | **0.102** | **±** | **0.011** | **15** | **0.56** | **-0.701** | **-1.274** |
|  | STA | 24 | 1 | 0 | **-** | 1 | 0.0008 | 0.0037 | 0.0000 | **-** | 0.009 | **±** | 0.009 | 0.020 | **±** | 0.020 | 2 | 0.39 | 0.776 | - |
|  | BAY | 24 | 1 | 0 | **-** | 1 | 0.0006 | 0.0028 | 0.0000 | **-** | 0.018 | **±** | 0.018 | 0.015 | **±** | 0.015 | 2 | 0.29 | 0.139 | - |
|  | GRO | 24 | 11 | 2 | **-** | 13 | 0.0082 | 0.0321 | 0.0013 | **-** | 0.162 | **±** | 0.027 | 0.193 | **±** | 0.037 | 5 | 0.60 | 0.167 | -0.325 |
|  | BRE | 22 | 2 | 1 | **-** | 3 | 0.0017 | 0.0065 | 0.0003 | **-** | 0.048 | **±** | 0.030 | 0.039 | **±** | 0.024 | 3 | 0.39 | 0.541 | -1.162 |
|  | INN | 22 | 11 | 4 | **-** | 15 | 0.0083 | 0.0269 | 0.0029 | **-** | 0.215 | **±** | 0.032 | 0.194 | **±** | 0.030 | 5 | 0.71 | -0.495 | -0.149 |
|  | KAL | 24 | 9 | 3 | **-** | 12 | 0.0064 | 0.0237 | 0.0014 | **-** | 0.189 | **±** | 0.048 | 0.151 | **±** | 0.036 | 4 | 0.43 | -0.235 | -0.916 |
|  | ARL | 24 | 9 | 2 | **-** | 11 | 0.0028 | 0.0108 | 0.0005 | **-** | 0.070 | **±** | 0.026 | 0.065 | **±** | 0.023 | 4 | 0.57 | -1.740 | -1.515 |
|  | GOTH | 22 | 11 | 4 | **-** | 15 | 0.0059 | 0.0213 | 0.0015 | **-** | 0.158 | **±** | 0.034 | 0.139 | **±** | 0.027 | 5 | 0.64 | -1.122 | -1.457 |

# Supplementary Figures

## Supplementary Figure 1

The pairwise linkage disequilibrium (LD) among the 7 SNPs in a 600kb region on chromosome arm 2R in the Selection and Control lines, and in the (combined) Field lines. The SNPs in the graphical table and the LD-plots are arranged from left to right, according to their positions on chromosome 2R (see Figure 1). The left column summarizes three estimators of LD: the raw (D) and scaled (D') estimates of frequency differences between the observed and expected numbers of SNP allele pairs, and the correlation coefficient between the SNPs (r). The color-coding relates to the level of significance (*P*-value, red for *P* < 0.01, yellow for non-significance), as assessed from the Chi-square statistic for marker independence (X^2), calculated for the scaled estimate D'. The right column provides a graphical representation of LD for the pairwise estimates of D' for each SNP. The line for the SNP in *mbl* is highlighted in blue.

# Supplementary Data Sheet

## Supplementary Data Sheet 1

The raw data for the genotypes and phenotypes of all individuals analyzed in this study. We scored the ability of individual *D. melanogaster* larvae from Selection and Control lines (144 individuals in total) and for Field lines (400 individuals in total), to resist parasitization by *A. tabida*. Eleven SNPs were genotyped in these same individuals. The Data Sheet provides the individual genotypes and phenotypes of all larvae, in two separate worksheets for the Selection and Control lines (GenoPheno_CS) and for the Field lines (GenoPheno_FL). These data form the basis for the analysis of individual genotype-phenotype associations and the linkage disequilibrium analysis, as published in the manuscript. In addition, to analyze the SNP frequencies throughout the selection procedure, we genotyped of a subset of 24 females from each Control and Selection line after 0 (source population), 1, 2, 3 and 5 generations of selection. The individual genotypes for all individuals are provided in a separate worksheet (Generations_CS).
