# Supplementary figures and images for: Natural and Artificial Selection for Parasitoid Resistance in Drosophila melanogaster Leave Different Genetic Signatures
Source: Front Genet. 2019 May 31;10:479. doi: 10.3389/fgene.2019.00479 (PMC6557190; doi:10.3389/fgene.2019.00479)

Selection lines

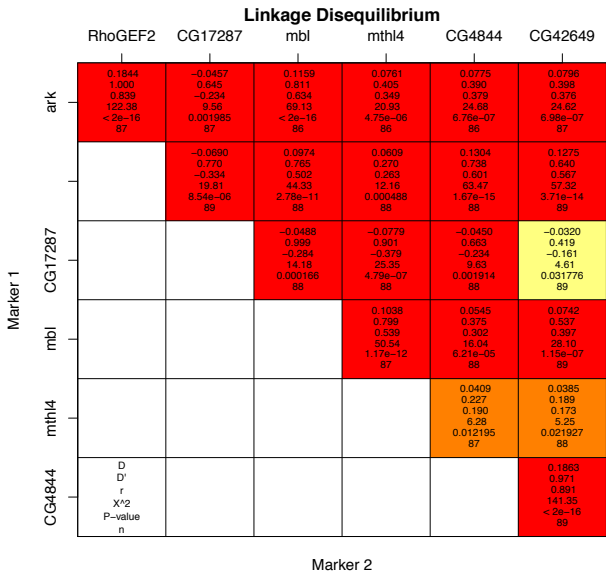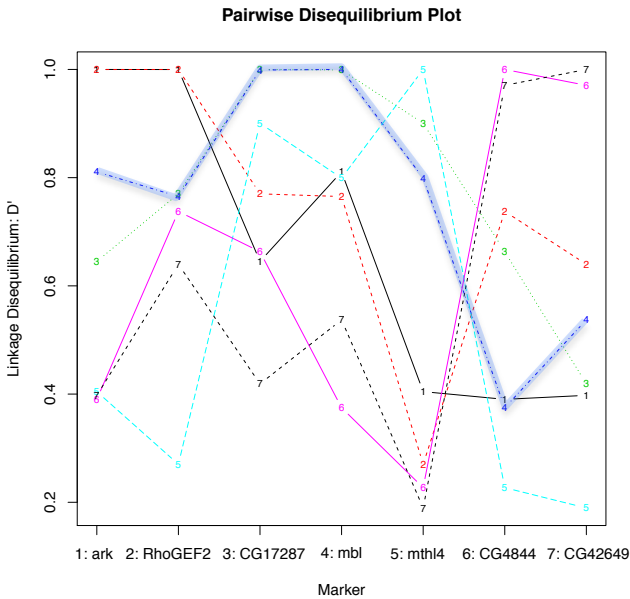

Control lines

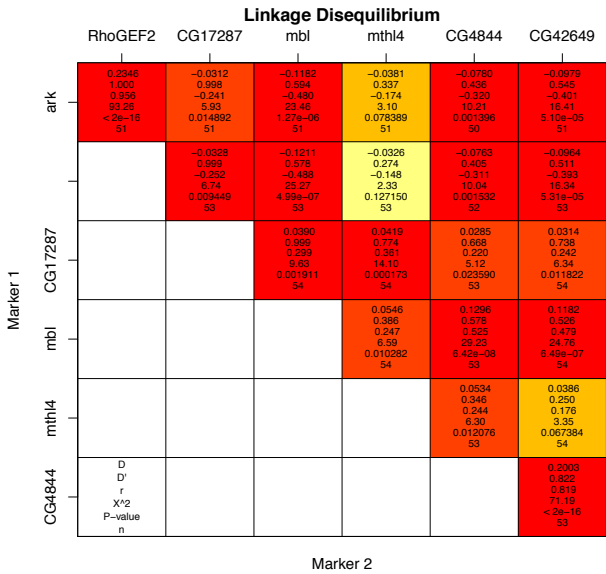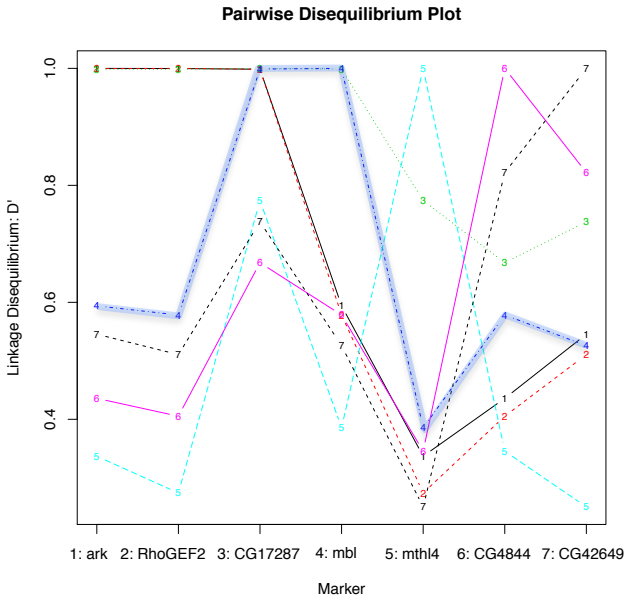

Field lines

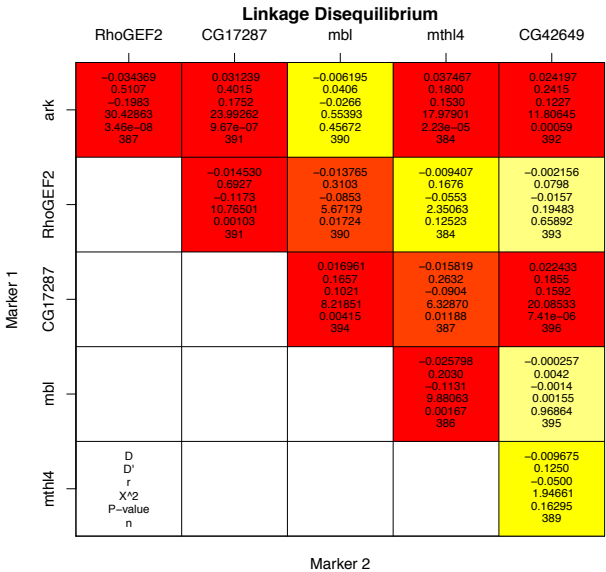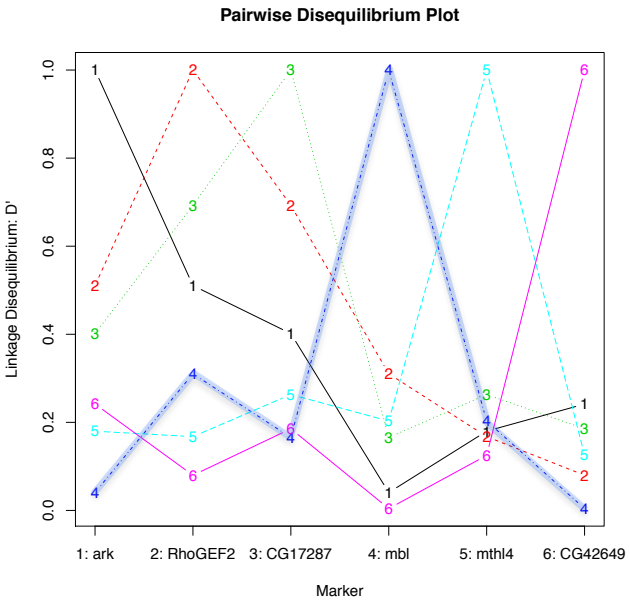

Supplement: Supplementary file 3 [file Image_1.pdf]
